# Nuclear Export of Pre-Ribosomal Subunits Requires Dbp5, but Not as an RNA-Helicase as for mRNA Export

**DOI:** 10.1371/journal.pone.0149571

**Published:** 2016-02-12

**Authors:** Bettina Neumann, Haijia Wu, Alexandra Hackmann, Heike Krebber

**Affiliations:** Abteilung für Molekulare Genetik, Institut für Mikrobiologie und Genetik, Göttinger Zentrum für Molekulare Biowissenschaften (GZMB), Georg-August Universität Göttingen, Germany; University of Toronto, CANADA

## Abstract

The DEAD-box RNA-helicase Dbp5/Rat8 is known for its function in nuclear mRNA export, where it displaces the export receptor Mex67 from the mRNA at the cytoplasmic side of the nuclear pore complex (NPC). Here we show that Dbp5 is also required for the nuclear export of both pre-ribosomal subunits. Yeast temperature-sensitive *dbp5* mutants accumulate both ribosomal particles in their nuclei. Furthermore, Dbp5 genetically and physically interacts with known ribosomal transport factors such as Nmd3. Similar to mRNA export we show that also for ribosomal transport Dbp5 is required at the cytoplasmic side of the NPC. However, unlike its role in mRNA export, Dbp5 does not seem to undergo its ATPase cycle for this function, as ATPase-deficient *dbp5* mutants that selectively inhibit mRNA export do not affect ribosomal transport. Furthermore, mutants of *GLE1*, the ATPase stimulating factor of Dbp5, show no major ribosomal export defects. Consequently, while Dbp5 uses its ATPase cycle to displace the export receptor Mex67 from the translocated mRNAs, Mex67 remains bound to ribosomal subunits upon transit to the cytoplasm, where it is detectable on translating ribosomes. Therefore, we propose a model, in which Dbp5 supports ribosomal transport by capturing ribosomal subunits upon their cytoplasmic appearance at the NPC, possibly by binding export factors such as Mex67. Thus, our findings reveal that although different ribonucleoparticles, mRNAs and pre-ribosomal subunits, use shared export factors, they utilize different transport mechanisms.

## Introduction

The biogenesis of eukaryotic ribosomal subunits begins in the nucleolus with the generation of a common 35S precursor transcript, which is processed by a series of cleavage events into the mature 18S, 25S and 5.8S rRNAs [1]. Already during transcription, various assembly factors and ribosomal proteins associate with the 35S pre-rRNA to form the 90S precursor particle that is subsequently split into a pre-60S and a pre-40S subunit. Upon completion of their nuclear biogenesis, different export receptors bind to distinct sites of the pre-ribosomal subunits to allow their passage through the hydrophobic meshwork of the nuclear pore complex (NPC) into the cytoplasm. The pre-60S particle interacts with the nuclear export signal (NES)-containing adaptor protein Nmd3 in *Saccharomyces cerevisiae* (human NMD3), which recruits the export receptor Xpo1 (CRM1/Exportin1) [2–5]. Xpo1 utilizes the Ran GTPase cycle to facilitate the directional transport of cargo through the NPC [6]. Both, Xpo1 and the Ran GTPase system are also involved in the export of the pre-40S subunit [7]. A possible NES-containing adaptor protein, however, is currently unknown, but among others Rio2 (hRio2) was discussed as a candidate [8]. Furthermore, the well-established mRNA export receptor heterodimer Mex67-Mtr2 (TAP-p15), which directly contacts the rRNA, is necessary for the transport of both pre-ribosomal particles [9, 10]. While less is known about additional export factors for the transport of the small pre-ribosomal subunit, Npl3, Bud20, Arx1 and Ecm1 were identified as auxiliary export factors for the pre-60S subunit [1]. Upon passage through the NPC, further maturation steps including the final processing of the 20S pre-rRNA to the mature 18S rRNA occur in the cytoplasm, before the ribosomal subunits are competent for translation initiation [11].

The DEAD-box RNA-helicase Dbp5 (Rat8/human DDX19) is well known for its essential function in mRNA export from the nucleus to the cytoplasm [12, 13]. Located at the cytoplasmic filaments of the NPC, Dbp5 was suggested to remodel emerging messenger ribonucleoparticles (mRNPs) and to dissociate bound transport factors such as Mex67 or Nab2 from the mRNA, which provides directionality in the transport process [14, 15]. For this function, Dbp5 undergoes an ATPase cycle, in which ATP-bound Dbp5 binds to the mRNPs and ADP-bound Dbp5 releases the mRNA and bound export factors [16]. The ATP-hydrolysis is stimulated by the cofactors Gle1 and IP_6_ (inositol hexakisphosphate) and the subsequent nucleotide exchange requires the nucleoporin Nup159/Rat7, which also attaches Dbp5 to the cytoplasmic side of the NPC [17–19]. A second essential role of Dbp5 and Gle1 was identified in translation termination in the cytoplasm [20, 21].

Here, we show that Dbp5 is required for the nuclear export of both the pre-40S and pre-60S ribosomal subunits. Interestingly, while the ATPase-dependent helicase activity of Dbp5 seems to be essential for mRNA export to displace bound export factors such as Mex67 from the emerging mRNA, our studies suggest that it is dispensable for the transport of ribosomal particles. Consequently, Mex67 is not displaced and remains bound until the ribosomes are engaged in translation. These results suggest that different RNPs require different transport mechanisms both involving Dbp5.

## Materials and Methods

### Yeast strains and plasmids

All yeast strains, plasmids and oligonucleotides used in this study are listed in the S1, S2 and S3 Tables, respectively.

Plasmid pHK1349 was created by amplification of the *RPL11B* ORF + 900bp upstream of the start codon by the oligonucleotides HK1485 and HK1486 from genomic yeast DNA. The PCR-fragment and pHK12 were digested with *Sac*II and *Xho*I and subsequently ligated.

pHK653 was digested with *Xba*I and *Sac*I and the resulting fragment was inserted into pHK87 by using the same restriction sites resulting in pHK666.

pHK1288 was created by digestion of pHK1288 (pGEX-6P-1) and pHK1282 (pGEX-4T-1-*DBP5*) with *Eco*RI and *Xho*I and ligation of *DBP5* in the pGEX-6P-1 backbone.

pHK789 was generated by amplification of the *ARX1* ORF + 1000bp upstream of the start codon by HK558 and HK562 from genomic yeast DNA and insertion in the pGEM-T plasmid (Promega). The resulting vector was digested with *Sac*II and *Xho*I and the fragment inserted into the *Sac*II and *Xho*I digested pHK12.

The strains HKY1356 and HKY1369—HKY1372 were generated by crossing HKY894 with HKY456 and HKY734 with HKY128, respectively.

HKY128 was transformed with pHK629 (*2μ RAT8 URA3*) and screened for the loss of the *CEN rat8-3 LEU2* plasmid to generate HKY456.

The diploid strain Y25036 from Euroscarf was transformed with pHK707 (*CEN mtr2-33 LEU2*) and after sporulation the haploid spores were selected for Geneticin resistance (*mtr2*::*kanMX4*) and the maintenance of the plasmid to obtain the strain HKY734.

The strain HKY90 was created by crossing the strain AG157.206A (*rat8-1*) [13] into the S288C background.

The strain HKY130 was crossed with the strain *rpb1-1* [22] to obtain HKY462.

### Growth analyses

Cells were spotted in 10-fold serial dilutions onto different selective agar plates and grown for three days at the indicated semi-permissive temperatures. To allow comparable growth on ura^-^ leu^-^ trp^-^ selective plates, all strains were transformed with plasmids containing the corresponding marker genes and temperature-sensitive alleles were rescued by the presence of the respective wild type genes on *URA3* containing plasmids. For growth analyses, cells were spotted onto FOA (5-Fluoroorotic Acid) containing plates to select for the loss of the covering wild type genes. The following strains have been used:

HKY36+pHK86+pHK87+pHK88 (WT), HKY894+pHK87 (*nmd3-2*), HKY456+pHK638+pHK86 (*rat8-3*), HKY1356+pHK638 (*rat8-3 nmd3-2*), HKY456+pHK655+pHK86 (*rat8-2*), HKY1356+pHK655 (*rat8-2 nmd3-2*), HKY1370+pHK87+pHK88 (WT), HKY1371+pHK86+pHK88 (*rat8-3*), HKY1372 (*mtr2-33*), HKY1369+pHK706 (*rat8-3 mtr2-33*).

### Sucrose-density fractionation experiments

The preparation and fractionation of sucrose-density gradients was essentially carried out following the protocols published previously [20, 23]. Briefly, yeast cell cultures of 200 ml were grown over night at 25°C to log-phase and then shifted for 1 h to the indicated temperatures. Cycloheximide (Roth) was added to a final concentration of 100 μg/ml and cells were incubated for 15 min on ice. After harvesting, the cell pellets were lysed with the same amount of glass beads in a FastPrep-24 machine (MP Biomedical) in lysis buffer (20 mM HEPES-KOH pH7.5, 10 mM KCl, 25 mM MgCl_2_, 1 mM EGTA, 1 mM DTT, 100 μg/ml Cycloheximide) supplemented with Complete, EDTA-free protease inhibitor cocktail (Roche). The lysates were centrifuged twice for 10 min at 21000 x g and 4°C. If indicated, the lysates were treated with 0.25 mg/ml RNase A (AppliChem) or with 100 mM EDTA pH 8.0 for 20 min on ice. For protein analyses 10 OD_260nm_ units, for subsequent co-immunoprecipitations 25 OD_260nm_ units and for ribosomal profile analyses 7 OD_260nm_ units of lysates were loaded onto the top of linear 7–47% (w/v) sucrose gradients (20 mM HEPES-KOH pH7.5, 10 mM KCl, 2.5 mM MgCl_2_) poured with the Gradient Master machine (Biocomp) and centrifuged for 3 h at 40000 rpm and 4°C in a TH-641 rotor and Sorvall WX80 ultracentrifuge (Thermo Scientific). The gradients were fractionated with a density-gradient fractionator (Teledyne Isco) while the absorbance at 254 nm was documented.

Protein fractions were precipitated with 10% TCA and subjected to SDS-PAGE and Western blotting. To be able to load the whole gradient on one gel, some fractions were pooled as indicated.

For subsequent co-immunoprecipitations, the indicated fractions from three gradients per strain were pooled, diluted with PBSKMT buffer (1x PBS, 3 mM KCl, 2.5 mM MgCl_2_, 0.3% Triton-X 100), mixed with 10 μl slurry of BSA-blocked GFP-Trap_A beads (Chromotek) and incubated on a rotator for 4.5 h at 4°C. If indicated, the samples were treated with 0.1 mg/ml RNase A (AppliChem). Afterwards, the beads were washed six times with PBSKMT, mixed with 25 μl 2x SDS-sample buffer and analyzed by SDS-PAGE and Western blotting. As input control, 100 μl of the pooled fractions were precipitated with 10% TCA.

From the profiles of the EDTA treated cells, the 60S/40S ratio was determined from three independent experiments and the Fiji software (1.48s Java 1.6.0_65) was used to calculate the areas underneath the 40S and 60S peaks, respectively.

### Western blot analyses

Polyclonal rabbit antibodies against Rpl35 (kindly provided by M. Seedorf, dilution 1:5000), Hem15 and Aco1 (kindly provided by R. Lill, dilution 1:7000 and 1:2000 respectively), Mex67 (kindly provided by C. Dargemont, dilution 1:50000), Rio2 (kindly provided by K. Karbstein, dilution 1:2000), Dbp5 (dilution 1:1000) and Rps3 (dilution 1:1000) were used. GFP-tagged proteins were detected with an anti-GFP antibody (sc-8334; Santa Cruz, dilution 1:1000) and myc-tagged proteins with an anti-myc antibody (sc-789; Santa Cruz, dilution 1:750). Monoclonal mouse antibodies against Pab1 (Santa Cruz, dilution 1:1000) and GST (sc-138; Santa Cruz, dilution 1:2000) and secondary anti-rabbit IgG (H+L)-HRPO and anti-mouse IgG (H+L)-HRPO (Dianova) antibodies were used. The signals were detected with Amersham ECL Prime Western Blotting Detection Reagent (GE Healthcare) and the FUSION-SL chemiluminescence detection system (Peqlab) and the Western blot analyses were quantified using the Bio1D software (Peqlab).

### Fluorescence *in situ* hybridizations (FISH)

The experiments were performed essentially as described [24]. Digoxigenin (DIG)-labeled RNA probes were used for detection of 25S and 18S rRNAs. For probe synthesis, PCR templates with a T7 transcription site on the antisense strand were generated by using HK1138 + HK1139 (25S rRNA probe) and HK1140 + HK1141 (18S rRNA probe). The antisense RNA probes were produced by *in vitro* transcription of the purified PCR templates with T7-RNA-polymerase (Thermo Scientific) and the DIG-UTP RNA labeling mix (Roche). A Cy3-end labeled oligo(dT)_50_ probe was used for detection of poly(A)^+^RNAs. If indicated, 25S rRNAs were detected with the Cy3-labeled oligonucleotide HK2200.

**Fig 1:**
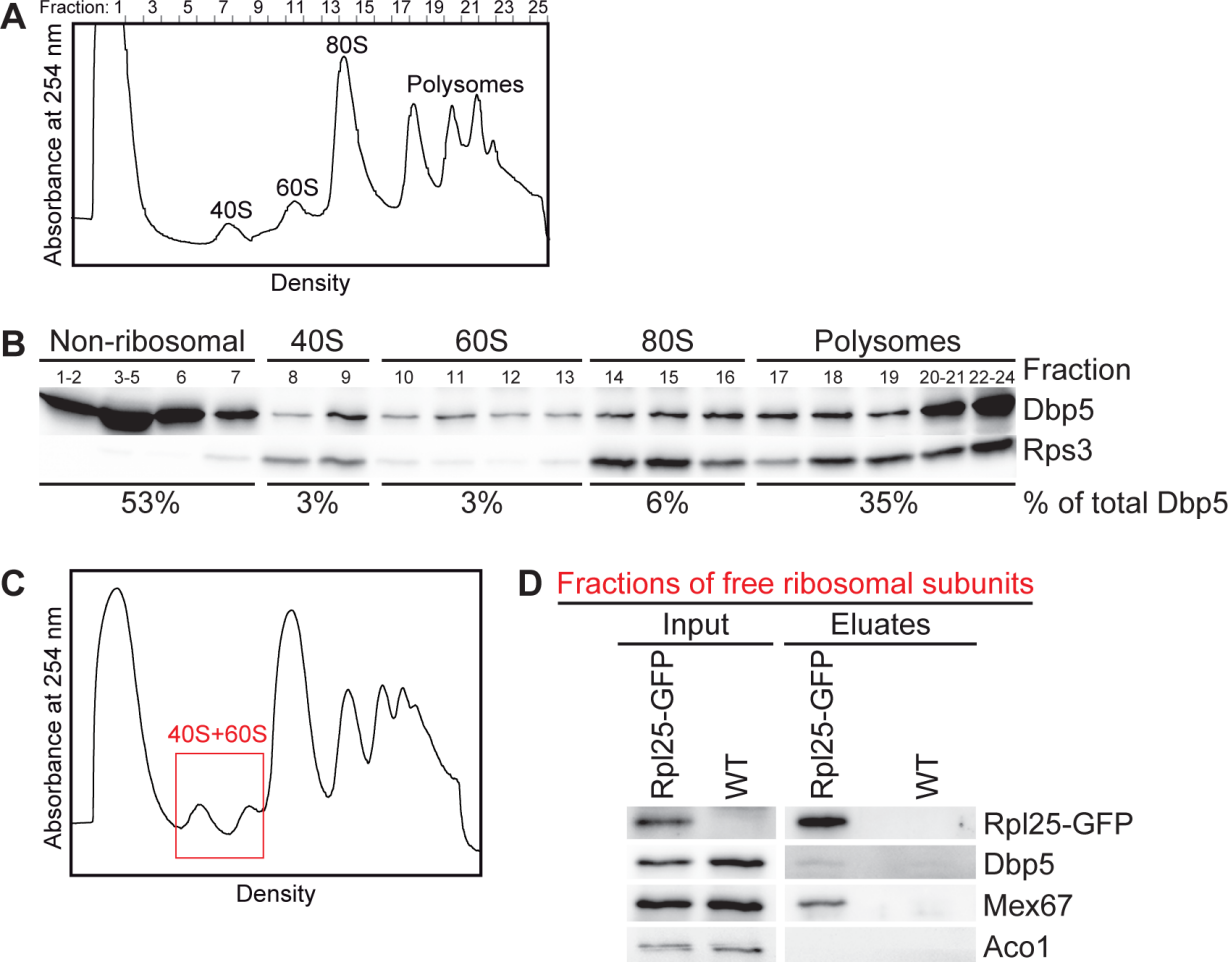
Dbp5 is associated with polysomes and both ribosomal subunits. **(A-B)** Wild type yeast lysates separated in sucrose-density gradients were fractionated while measuring the absorbance at 254 nm (A_254nm_) that results in the displayed profile **(A)**. The corresponding separated protein fractions are shown in Western blot analyses with antibodies against Dbp5 and the ribosomal protein Rps3 **(B)**. The percentage of the total cellular amount of Dbp5 in the non-ribosomal, 40S, 60S, 80S and polysomal fractions is indicated. **(C-D)** Western blot analyses of Rpl25-GFP immunoprecipitations from gradient fractions of the free ribosomal subunits show co-precipitation of Dbp5. Wild type yeast lysates were separated in sucrose-density gradients and upon fractionation, the free ribosomal subunits containing fractions (red framed area) were subjected to co-immunoprecipitation experiments. The correct fractions were chosen according the flow through photometry profiles displayed in **(C)**. Detection of Mex67 served as positive control and the mitochondrial protein Aco1 served as a negative control.

Log-phase cells were shifted for 1 h to the indicated temperatures and then fixed with 4% formaldehyde at room temperature followed by a zymolyase treatment. Spheroplasts were permeabilized with P-solution (see below)/0.5% triton X-100 and then pre-hybridized with Hyb-mix (50% formamide, 5x SSC, 1x Denhardts, 10 μg/ml heparin) supplemented with 500 μg/ml of tRNAs and 500 μg/ml of denatured *Salmon sperm*-DNA for 1 h. Cells were further treated with DIG-labeled RNA probes, Cy3-labeled oligo-25S or Cy3-labeled oligo(dT)_50_ probes at 37°C overnight followed by washing once with 2x SSC, once with 1x SSC, once with 0.5x SSC at 37°C and once with 0.5x SSC at 25°C (30 min per washing step). DIG was detected with a sheep anti-Digoxigenin Fab-FITC antibody (Roche) diluted 1:200 in ABB (1x PBS, 10% heat-inactivated FCS, 0.3% tween-20). Cell nuclei were stained with Hoechst 33342. Microscopy was performed with a Leica AF6000 microscope and pictures were taken with a Hamamatsu 1394 ORCA-ERA camera and the LAS AF 1.6.2 software (Leica).

A magnification of a representative cell is shown for each strain and the amount of cells revealing similar nuclear accumulation of the fluorescent signal is indicated. The fluorescent signals of the whole cell and of its nucleus of at least 10 cells per strain from at least three independent experiments were quantified by using the Fiji software (1.48s Java 1.6.0_65). Subsequently, the intensity of the nuclear signal was related to that of the complete cell and to the ratio of wild type to calculate the average fold enrichment of the nuclear signal.

### GFP-microscopy

Mid-log phase yeast cells were shifted for 1 h to their non-permissive temperatures. Afterwards, the cells were briefly fixed with 2.6% formaldehyde, immediately centrifuged for 5 min at 2050 x g and 4°C and washed with 0.1 M phosphate-buffer and with P-solution (0.1 M phosphate-buffer pH6.5, 1.2 M sorbitol). After transfer on polylysine (Sigma Aldrich)-coated microscope slides, the cells were permeabilized by treatment with 0.5% TritonX-100/P-solution and the DNA was stained with Hoechst 33342. Microscopy studies were performed as described in “Fluorescence *in situ* hybridizations”.

### Immunofluorescence

The experiments were performed as described previously [25]. Yeast cells were grown to mid-log phase, shifted for 1 h to their restrictive temperatures and fixed with 4% formaldehyde for 1 h at room temperature. After zymolyase treatment, spheroplasts were transferred to polylysine-coated microscope slides, permeabilized with 0.5% triton X-100/P-solution (0.1 M phosphate-buffer pH6.5, 1.2 M sorbitol), blocked with antibody blocking buffer ABB (1x PBS, 10% heat-inactivated FCS, 0.3% tween-20) for 1 h at room temperature and incubated with anti-Nop1 antibodies diluted 1:2500 in ABB overnight at 4°C. Following four times washing with ABB, anti-rabbit Alexa Flour 594 secondary antibody (from Invitrogen) diluted 1:100 in ABB was added for 2 h at room temperature. After washing with ABB and 0.1% Tween-20/PBS, the DNA was stained with Hoechst 33342 and microscopy studies were performed as described in “Fluorescence *in situ* hybridizations”.

For co-localization of 25S rRNAs and Nop1 proteins, the *in situ* hybridization protocol was performed first with the Cy3-labeled oligonucleotide HK2200 against 25S rRNAs followed by the immunofluorescence protocol with anti-Nop1 primary antibodies and anti-rabbit Alex Fluor 488 secondary antibodies (from Invitrogen).

### Northern blotting

DIG-labeled RNA probes against the 25S rRNA (HK1138 + HK1139), 18S rRNA (HK1140 + HK1141), 20S, 33S and 35S pre-rRNAs (HK1893 + HK1894), 27S, 33S and 35S pre-rRNAs (HK1895 + HK974) and U2 snRNA (HK1723 + HK1724) were synthesized as described in “Fluorescence *in situ* hybridizations”.

Indicated strains were shifted for 1 h to their restrictive temperatures and total RNA from mid-log yeast cells was extracted by using the kit “NucleoSpin RNA” (Macherey-Nagel) following the protocol of the manufacturer. 5 μl of RNA solution in DEPC-water containing 1 μg of total RNA were mixed with 10 μl RNA-Loading Dye (50% deionized formamide, 6% Formaldehyde, 1x MOPS, 25 ng/ml Ethidium bromide, 10% Glycerol, Bromophenol blue and Xylene cyanol), denatured for 10 min at 65°C and electrophoresed on a 1% agarose/MOPS gel containing 2% formaldehyde in running buffer (1x MOPS) at 80V for 4–5 h. After washing the gel once in DEPC-water and twice in 20x SSC for 15 min, the RNA was transferred onto a positively charged nylon membrane (Amersham Hybond-N^+^). RNA was crosslinked by exposing the membrane for 7 min to UV light (5000 mJ) and incubation for 2 h at 80°C. After pre-hybridization for 1 h at 68°C with hybridization buffer (0.5 M Na-Phosphate pH7.2, 7% SDS, 1 mM EDTA), 1 μl of each DIG-labeled RNA probe was added and hybridized overnight at 68°C. The membranes were subsequently washed four times for each 15 min in 2x SSC/ 0.1% SDS at room temperature, 1x SSC/ 0.1% SDS at room temperature and twice in 0.5x SSC/ 0.1% SDS at 68°C. DIG was detected with anti-Digoxigenin-AP, Fab fragments (Roche) and as substrate CSPD (Roche) following the protocol of the manufacturer on X-ray films.

### Co-immunoprecipitation experiments

The experiments were performed as described previously [23]. Yeast cells were grown in 300–500 ml selective media to mid-log phase and if required, shifted for 1h to the indicated temperatures. After harvesting, the cell pellets were lysed with the same amount of glass beads in a FastPrep-24 machine (MP Biomedical) in PBSKMT (1x PBS, 3 mM KCl, 2.5 mM MgCl_2_, 0.5% Triton-X 100) supplemented with protease inhibitor cocktail for yeast (Sigma-Aldrich) and Complete, EDTA-free protease inhibitor cocktail (Roche). After two times centrifugation for 10 min at 21000 x g and 4°C, 500–1000 μl of the clarified lysate were incubated with 10 μl slurry of GFP-Trap_A beads (Chromotek) for 3 h rotating at 4°C. If indicated, the samples were treated with 0.2 mg/ml RNase A (AppliChem) for 30 min at 4°C. The beads were washed six times with PBSKMT and mixed with 25 μl 2x SDS-sample buffer. After boiling, the eluted proteins were separated on 10% SDS-polyacrylamide gels and analyzed by Western blotting.

### *In vitro*-binding studies

His-Mtr2 and Mex67 were co-expressed in *E*. *coli* BL21 DE3 cells and the heterodimer was purified by affinity chromatography with Protino Ni-NTA agarose (Macherey-Nagel) and stored at -80°C in elution buffer (30 mM HEPES pH7.5, 100 mM NaCl, 10% glycerol, 250 mM imidazole). GST-Dbp5 and GST, respectively, were expressed in *E*. *coli* Rosetta 2 cells and purified by affinity chromatography with GSTrap 4B Glutathione Sepharose (GE Healthcare) and stored at -80°C in elution buffer (for GST-Dbp5: 50 mM Tris pH 7.5, 150 mM NaCl, 30 mM Glutathione reduced; for GST: 30 mM HEPES pH 6.5, 100 mM NaCl, 10% glycerol, 2 mM DTT, 30 mM Glutathione reduced).

For binding studies, 100 μg of purified GST-Dbp5 or 100 μg of GST were mixed with 100 μl of bacterial lysates and incubated with 10 μl slurry of Glutathione Sepharose 4B beads (GE Healthcare) in lysis buffer (20 mM HEPES pH7.5, 100 mM NaCl, 4 mM MgCl_2_, 10% Glycerol, 0.1% NP-40, 1 mM DTT, protease inhibitor Complete from Roche) for 1 h rotating at 4°C. The lysis buffer was supplemented with 1 mM ATP and/or 0.2 mg/ml RNase A (AppliChem) if indicated. The beads were washed once with lysis buffer. Subsequently, 28 μg of purified His-Mtr2+Mex67 was added and incubated for 1 h at 4°C. Afterwards, the beads were washed six times with lysis buffer and bound proteins were eluted with 40 μl of 2x SDS-sample buffer. Half of the eluates and 14 μg of GST-Dbp5 / 16 μg of GST / 9 μg of His-Mtr2+Mex67 as input controls were subjected to SDS-PAGE and Western blotting analyses or Coomassie staining, respectively.

## Results

### Mutants of *DBP5* are defective in the nuclear export of both pre-ribosomal subunits

**Fig 2:**
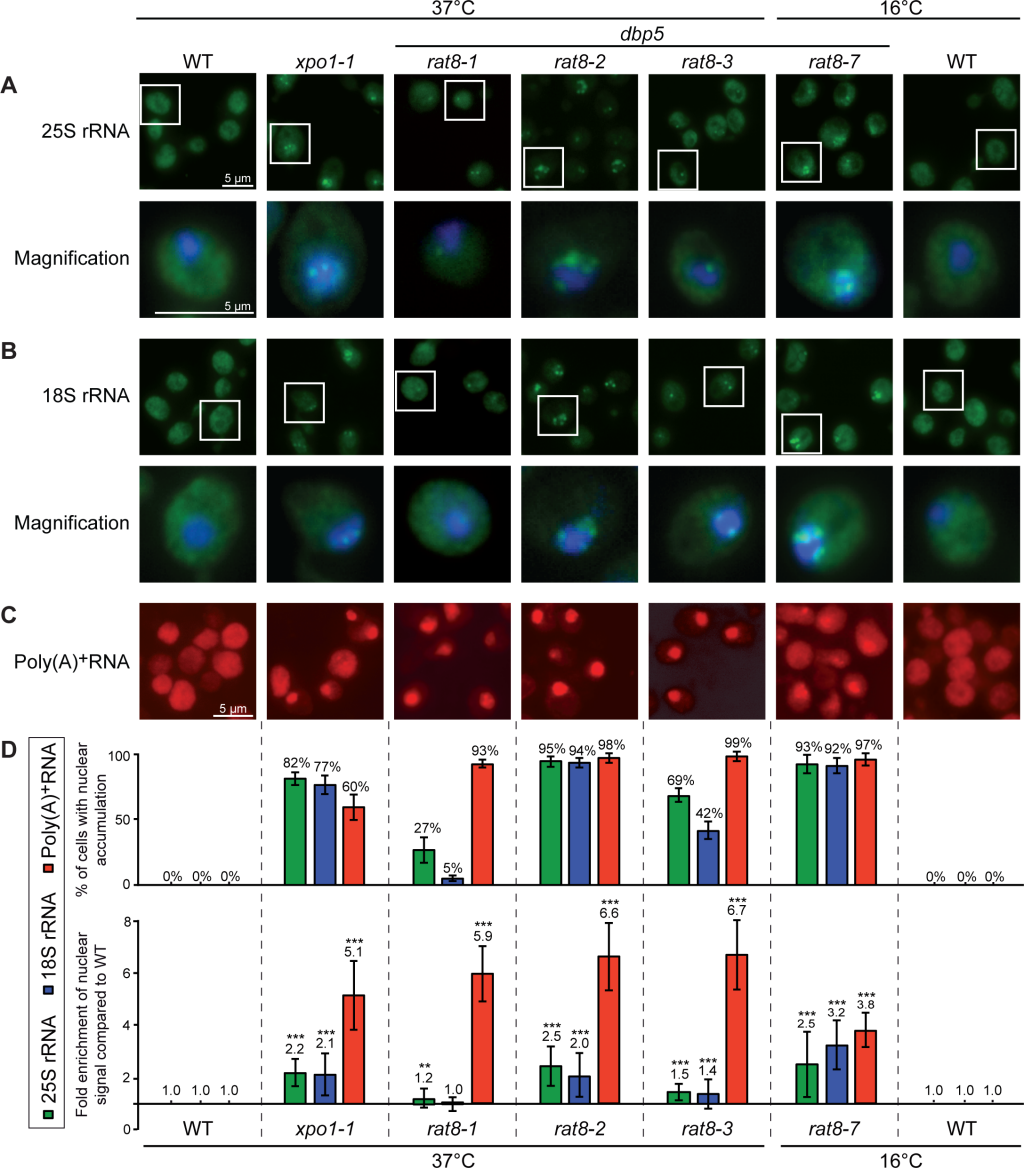
Mutants of *DBP5* are defective in the nuclear export of both pre-ribosomal subunits. Fluorescence *in situ* hybridization experiments with probes against the 25S rRNA **(A)**, the 18S rRNA **(B)** and poly(A)^+^RNA **(C)** reveal a nuclear accumulation of the fluorescent signal in different temperature-sensitive *dbp5/rat8* mutants and in a mutant of the karyopherin Xpo1/Crm1 (*xpo1-1*), which served as positive control, upon 1 h temperature shifts to their indicated non-permissive temperatures. The lower panels of **(A)** and **(B)** display a magnification of a representative cell, where the FITC channel was merged with the DNA signal. All single channels and merged pictures are shown in S2 Fig. (WT = wild type)**. (D)** Statistical analyses of **(A-C)**. The upper panel indicates the amount of cells with nuclear accumulation (error bars represent the standard deviation). The lower panel shows the average intensities of the nuclear signal of at least 10 cells relative to the whole cell and compared to wild type. Error bars represent the standard deviation and p-values were calculated by an unpaired students t-test (*** = p<0.001 and ** = p<0.01).

As expected for its function in translation termination, Dbp5 is associated with polysomes (Fig 1A and 1B and [20]). However, we noticed that Dbp5 is also present in the fractions of both free ribosomal subunits in sucrose-density gradient studies (Fig 1A and 1B) and is weakly, but specifically detectable in immunoprecipitations of the large ribosomal protein Rpl25 from these fractions (Fig 1C and 1D). These results suggest that Dbp5 is already transiently associated with free ribosomal subunits and might have a function on the subunits prior to translation. Considering that this RNA-helicase is necessary for the directional transport of mRNAs into the cytoplasm [12, 13], we investigated if Dbp5 would also be required for the nuclear export of pre-ribosomal subunits. Indeed, fluorescence *in situ* hybridization (FISH) experiments with fluorescent 25S and 18S rRNA probes revealed that almost all cells of the temperature-sensitive *dbp5* mutants *rat8-2* and *rat8-7* accumulate pre-60S and pre-40S subunits in their nuclei upon shift to their restrictive temperatures (Fig 2A and 2B; for all split channels and merged pictures see S1A and S1B Fig). These defects are comparable with those seen in the established export receptor mutant *xpo1-1* (Fig 2D) indicating that Dbp5 is required for the nuclear export of both pre-ribosomal subunits. Interestingly, even though also detectable, the effect is less strong and affects fewer cells in *rat8-3*, while such defects are only barely visible in *rat8-1* cells (Fig 2A and 2B and 2D). Likewise, all of these mutants reveal a nuclear accumulation of GFP-tagged Rpl25 and Nmd3 as established reporter proteins for the large [3] and Rps2-GFP as marker for the small ribosomal subunit [26] mirroring the effect seen with the rRNA probes (S2 Fig). In support of our data, *rat8* mutants were identified earlier in a screen for mutants that mislocalize another reporter protein of the large ribosomal subunit, Rpl11-GFP, in their nuclei, however, this initial finding was not further characterized [27]. Together, these results show that Dbp5 is required for the nuclear export of pre-ribosomal subunits.

One might think that the ribosomal export defects could be a consequence of the mRNA export defects. However, the intensities of the defects in ribosomal transport do not correlate with the extent of the mRNA export defects in the different *dbp5* mutants (Fig 2C and 2D). While *rat8-2* and *rat8-7* cells both have very strong ribosomal subunit localization defects, their mRNA export defects differ and particularly *rat8-7* has the weakest mRNA transport defect of all tested *dbp5* mutants. *Vice versa*, the ribosomal transport defects seen in *rat8-1* and *rat8-3* are rather mild, but their mRNA export is strongly impaired. Thus, the missing correlation between mRNA and ribosomal transport defects in all *dbp5* mutants suggests that the observed ribosomal mislocalizations are not simply caused by defects in nuclear mRNA export. Furthermore, the onset of both, mRNA and ribosomal export defects is rapid and already detectable after short temperature shifts (S3 Fig and [13]), which further argues for a direct role of Dbp5 in ribosomal transport.

### Mutants of *DBP5* show no unique defects in ribosomal subunit biogenesis

**Fig 3:**
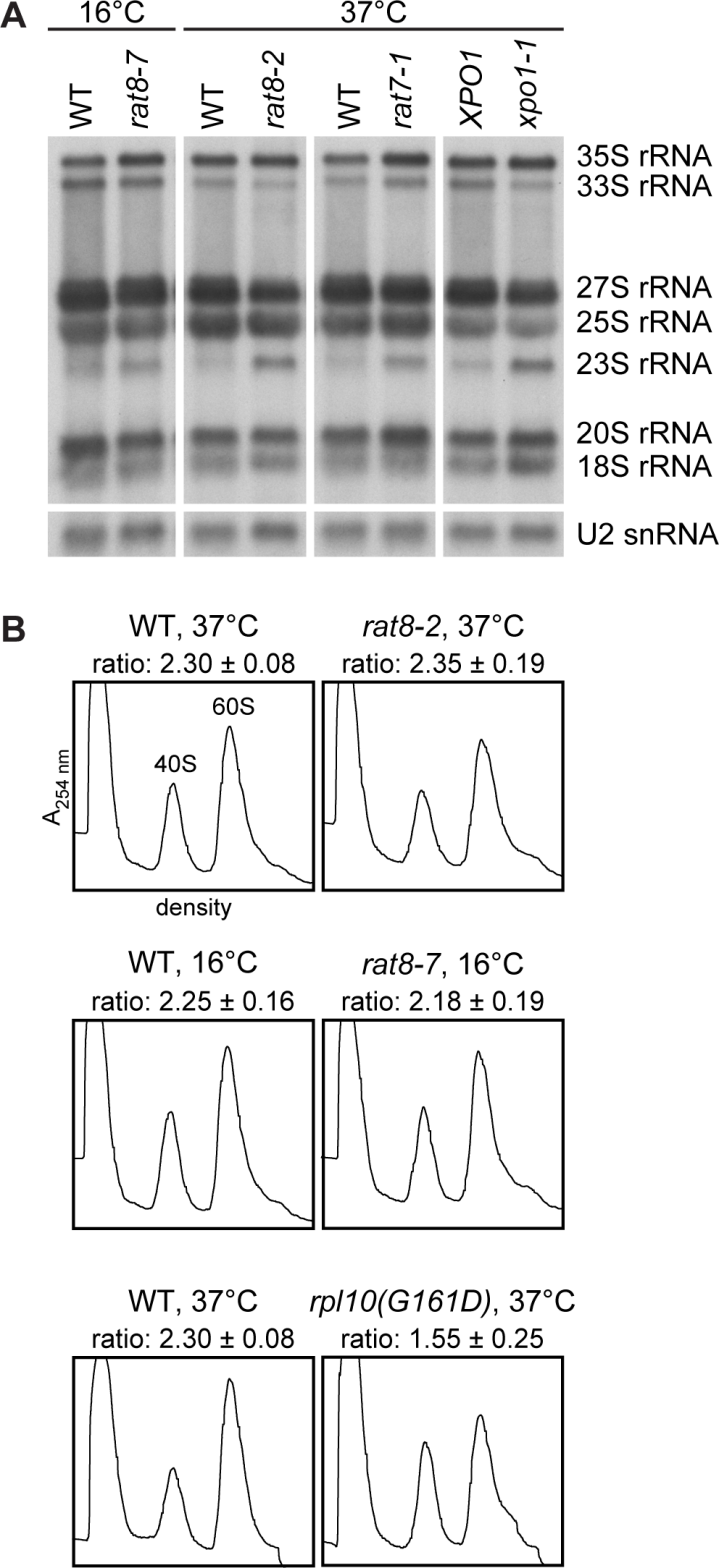
Mutants of *DBP5* show no unique defects in the biogenesis of ribosomal subunits. **(A)** The steady state levels of the different rRNAs and their precursors in the *dbp5* mutants *rat8-2* and *rat8-7* are comparable with those in the export mutants *rat7-1* and *xpo1-1*. Northern blot analyses with DIG-labeled RNA probes targeting the indicated rRNA species and U2 snRNAs (loading control) are shown. Total RNA of the different strains was extracted upon 1 h shifts to their restrictive temperatures and 1 **μ**g was separated by formaldehyde/MOPS-agarose gel electrophoresis. **(B)** The total amount of 40S and 60S subunits is not altered in *dbp5* mutants. Flow through photometry (A_254nm_ = absorbance at 254 nm) profiles of ultra-centrifuged sucrose gradients from *rat8-2* and *rat8-7* cells shifted to their indicated non-permissive temperatures and treated with 100 mM EDTA reveal no significant change of their peak sizes and the 60S to 40S subunit ratio in comparison to the wild type. In contrast, *rpl10(G161D)* served as a positive control of the assay and shows a decreased 60S peak.

To rule out that the observed defects in pre-ribosomal subunit export are caused by failures of their biogenesis, the steady state level of the different rRNAs and their precursor molecules was analyzed by Northern blotting in the strongest ribosomal transport mutants *rat8-2* and *rat8-7* (Fig 2). Indeed, only minor changes such as a slight increase in the 35S pre-rRNA levels and the appearance of an aberrant 23S rRNA are visible (Fig 3A). These variations are comparable to those of the temperature-sensitive mutants *rat7-1* and *xpo1-1* (Fig 3A and [7, 28, 29]), which have also defects in the nuclear export of both pre-ribosomal subunits and mRNAs [7, 27, 30]. Thus, slight defects in the rRNA processing could be a typical phenotype of nuclear export mutants and might be caused by the nuclear retention of pre-ribosomal particles and/or certain mRNAs.

In addition to that, we analyzed whether the production of ribosomal subunits in general is disturbed in the *dbp5* mutants and determined their overall amount of 40S and 60S subunits. All lysates were treated with 100 mM EDTA to disrupt the 80S ribosomes and to obtain free ribosomal subunits, which were separated in sucrose-density gradients to receive two distinct peaks representing the 40S and 60S subunits. Both peak areas were calculated and set into relation. As expected, wild type cells reveal a 60S:40S ratio of ~2.3 (Fig 3B) [30], while *rpl10(G161D)* cells defective in the cytoplasmic maturation of large ribosomal subunits [31] show a reduced 60S peak size and a decreased subunit ratio of 1.55 (Fig 3B). In contrast to the maturation mutant, the ratios of *rat8-2* and *rat8-7* cells are comparable to wild type. Moreover, the entire amounts of both subunits are unchanged, which is reflected by basically the same peak sizes as in wild type. These results indicate that the biogenesis of ribosomal subunits is not detectably affected in *dbp5* mutants. As the ribosomal export defects in *rat8-2* and *rat8-7* are rather strong (Fig 2A and 2B and 2D), it seems unlikely that potential slight processing defects cause the nuclear accumulation of ribosomal subunits.

### Dbp5 genetically and physically interacts with ribosomal export factors

**Fig 4:**
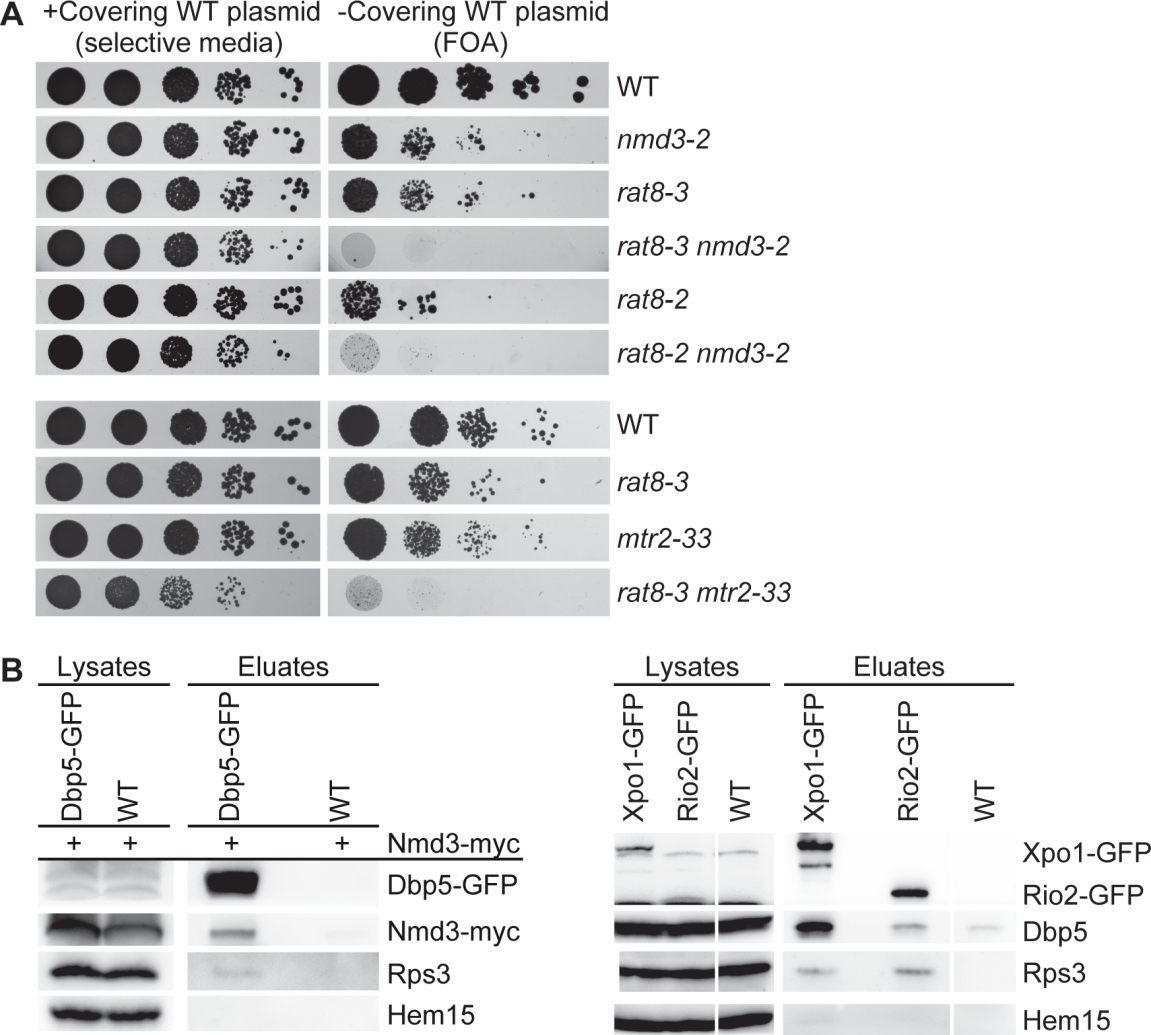
Genetic and physical interactions of Dbp5 and ribosomal export factors. **(A)** Dbp5 genetically interacts with the established ribosomal transport factors Nmd3 and Mtr2. The indicated strains were spotted in serial dilutions onto plates either selecting for a covering the respective wild type gene containing plasmid (selective media) or for the loss of this plasmid (FOA = 5-Fluoroorotic Acid). The strains *rat8-3* and *rat8-2* in combination with *nmd3-2* or *mtr2-33*, which has a specific export defect for ribosomal subunits, show synthetic growth defects after three days at 30°C. **(B)** Physical interactions of Dbp5 with Nmd3, Xpo1 and Rio2. Western blot analyses show co-precipitated Nmd3-myc in the Dbp5-GFP immunoprecipitation (left panel) and co-precipitated Dbp5 in the Xpo1-GFP and Rio2-GFP pull-downs (right panel). The *DBP5-GFP* expressing strain and the wild type (WT) were transformed with an *NMD3-13xmyc* containing plasmid. Detection of Hem15 served as a negative control and of Rps3 as a positive control.

Additional support for a function of Dbp5 in transporting pre-ribosomal subunits came from genetic analyses of *rat8-3* and *rat8-2* cells. Their combination with mutants of *NMD3* and *MTR2*, which are exclusively defective in the export of large ribosomal subunits, but not in mRNA transport [3, 32], result in synthetic lethality (Fig 4A). Interestingly, even the milder export mutant *rat8-3* shows the synthetic growth defect with *nmd3-2* and *mtr2-33*. These findings suggest that the mutations in these proteins might affect the same cellular process: The nuclear export of pre-ribosomal subunits. Remarkably, even though genetic interactions of *DBP5* with several mRNA processing and export factors were identified earlier [33–35], the synthetic lethality with ribosomal transport factors has not been uncovered before. Although the synthetic growth defects of the double mutants are quite severe, it remains possible that these effects are influenced by the mRNA export defects of the *dbp5* mutants. However, this seems rather unlikely, as for example *mtr2-21* mutants with exclusive mRNA transport defects show no strong genetic interaction with *nmd3-2*, whereas *mtr2-33* mutants with exclusive ribosomal transport defects are synthetic lethal with *nmd3-2* [32].

Thus, to gain further evidence for a role of Dbp5 in ribosomal export, we performed physical interaction studies with established ribosomal export factors. Supportingly, Dbp5 associates with Nmd3-, Rio2- and Xpo1-containing particles in co-immunoprecipitation experiments *in vivo* (Fig 4B and reciprocal co-immunoprecipitations are shown in S4A and S4B Fig) suggesting that Dbp5 physically contacts the export factor-bound pre-40S and pre-60 ribosomal subunits during nuclear export. Together, these genetic and in particular these physical interactions support a function of Dbp5 in ribosomal transport.

### Dbp5 awaits pre-ribosomal subunits at the cytoplasmic side of the NPC

**Fig 5:**
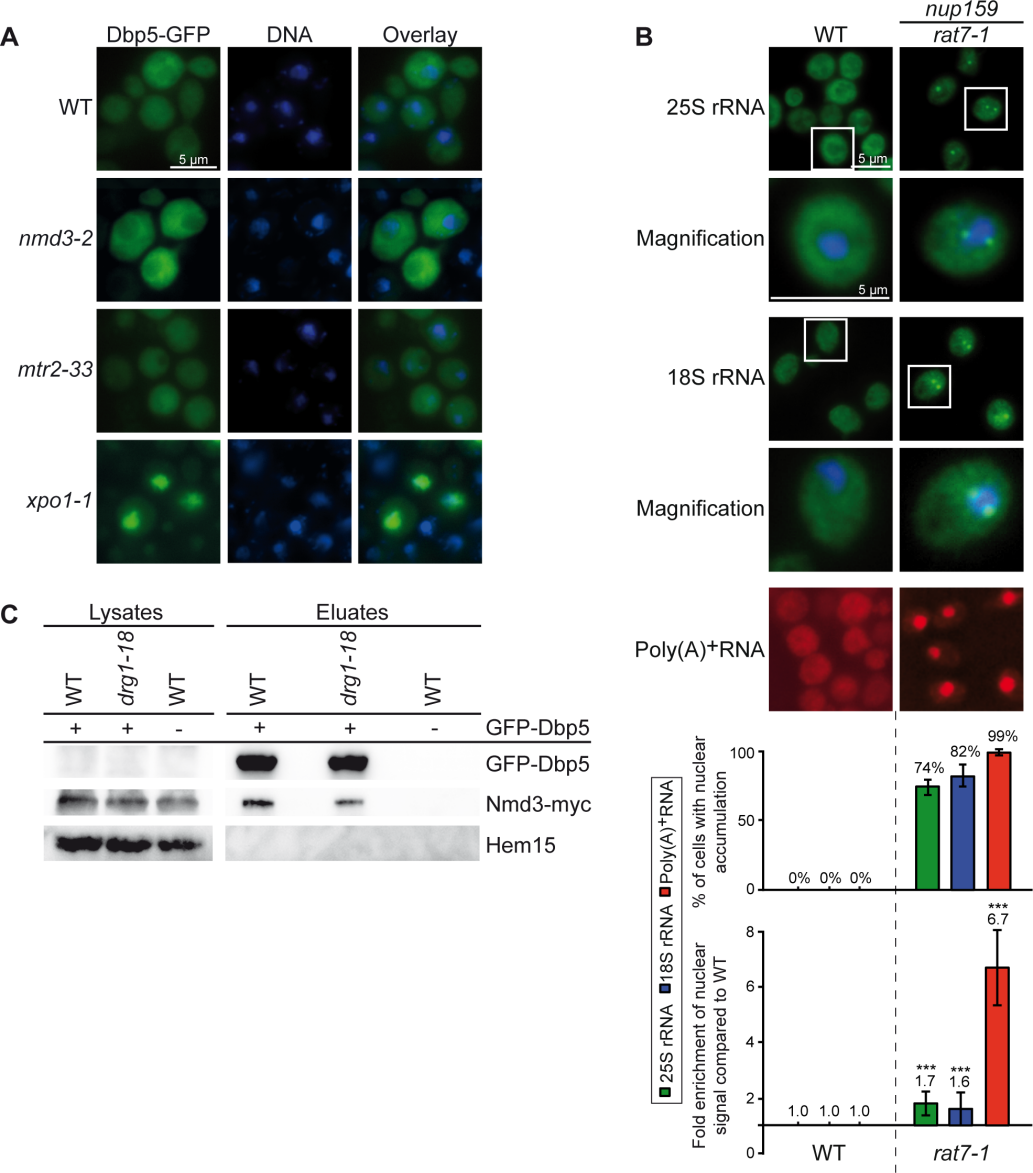
Dbp5 does not accompany ribosomal particles through the NPC, but seems to be required at its cytoplasmic side. **(A)** Dbp5 does not travel with the pre-ribosomal subunits from the nucleus to the cytoplasm. Fluorescence microscopy images of GFP-tagged Dbp5 show a cytoplasmic distribution in wild type, *mtr2-33* and *nmd3-2* cells upon shift to 37°C for 1 h. In contrast, GFP-Dbp5 accumulates in the nucleus of shifted *xpo1-1* cells. **(B)** *rat7-1* cells show mild ribosomal export defects. Fluorescence *in situ* hybridization experiments with probes against the 25S rRNA, 18S rRNA and poly(A)^+^RNA are shown in *rat7-1* and wild type cells upon shifts for 1 h to 37°C. Statistical analyses were performed as shown in Fig 2D. **(C)** The association of Nmd3-myc with GFP-Dbp5 is not increased in the *drg1-18* strain compared to wild type (WT). Western blot analyses of an immunoprecipitation experiment with GFP-Dbp5 and co-precipitated Nmd3-myc are shown upon 1 h shifts to 37°C. Detection of Hem15 served as non-binding control.

Similar to nuclear mRNA export, Dbp5 might also be needed for ribosomal transport at the cytoplasmic filaments of the NPC to remodel the arriving RNPs. However, it also seems possible that Dbp5 may travel together with the subunits from the nucleus into the cytoplasm. To distinguish between these possibilities, we investigated the localization of Dbp5-GFP in the *nmd3-2*, *mtr2-33* and *xpo1-1* strains defective in ribosomal transport (Fig 2A and 2B and [3, 32]). Interestingly, while Dbp5 accumulates in the nuclei of *xpo1-1* cells, it remains cytoplasmic in the *nmd3-2* and *mtr2-33* mutants like in wild type (Fig 5A). These results suggest that Dbp5 is not accompanying pre-ribosomal subunits through the NPC, but as reported earlier is actively exported from the nucleus by Xpo1 [36]. Thus, Dbp5 seems not to shuttle with pre-ribosomal subunits. It might rather act on the subunits from inside the nucleus or from the cytoplasmic side of the NPC.

If Dbp5 is required at the cytoplasmic exit site of the NPC to assist the export of pre-ribosomal subunits, the dissociation of the protein from its cytoplasmic NPC anchor Nup159/Rat7 [36–38] should result in decreased ribosomal transport. Therefore, we performed FISH experiments with *rat7-1* cells, in which Dbp5 is detached from the nuclear rim at the restrictive temperature [36]. Indeed, *rat7-1* cells show slight, but clearly detectable ribosomal export defects (Fig 5B; for all split channels and merged pictures see S5A Fig). Similar results were obtained for *rat7ΔN* cells (S5B Fig) specifically lacking the interaction domain for Dbp5 [36]. These defects might not be as strong as the defects seen in *dbp5* mutants (Fig 2D), because the presence of untethered Dbp5 in the cytoplasm of the *rat7* mutants might be sufficient to support the nuclear export of both pre-ribosomal subunits to some extent. Notably, the export defect of pre-ribosomal subunits present in *rat7-1* is considerably milder than that of mRNAs in this mutant, which might indicate differences in both transport pathways. Thus, rather the localization of Dbp5 at the NPC than the function of Nup159 as an ADP-release factor for Dbp5 [17] might be important for the export of pre-ribosomal subunits.

To support ribosomal transport, it is either possible that Dbp5 acts shortly from the cytoplasmic filaments, to which it remains associated, or that the protein is loaded onto the transported ribosomal particles at the NPC. In this case, Dbp5 might not be released until the subsequent cytoplasmic biogenesis steps occur, as described for shuttling maturation and export factors such as Arx1 and Nmd3. They accumulate on immature pre-60S particles in the cytoplasm of a temperature-sensitive mutant of *DRG1* (*drg1-18*), which prevents subsequent maturation steps ([39, 40] and S6 Fig). If this would also be the case for Dbp5, one would expect that the release defects seen in *drg1-18* should result in an increased binding of Dbp5 and Nmd3, which is not visible in co-immunoprecipitation studies (Fig 5C). In contrast, the interactions of Mex67 and Nmd3 with Arx1 are enhanced in *drg1-18* compared to wild type cells (S6 Fig), showing that the chosen conditions were sufficient to induce cytoplasmic accumulation of immature pre-60S subunits. These findings indicate that Dbp5 is not displaced from ribosomal particles at later cytoplasmic maturation steps, suggesting that the protein acts earlier, immediately upon the cytoplasmic appearance of the pre-ribosomal subunits at the NPC.

### The ATPase cycle of Dbp5 seems to be dispensable for ribosomal transport

**Fig 6:**
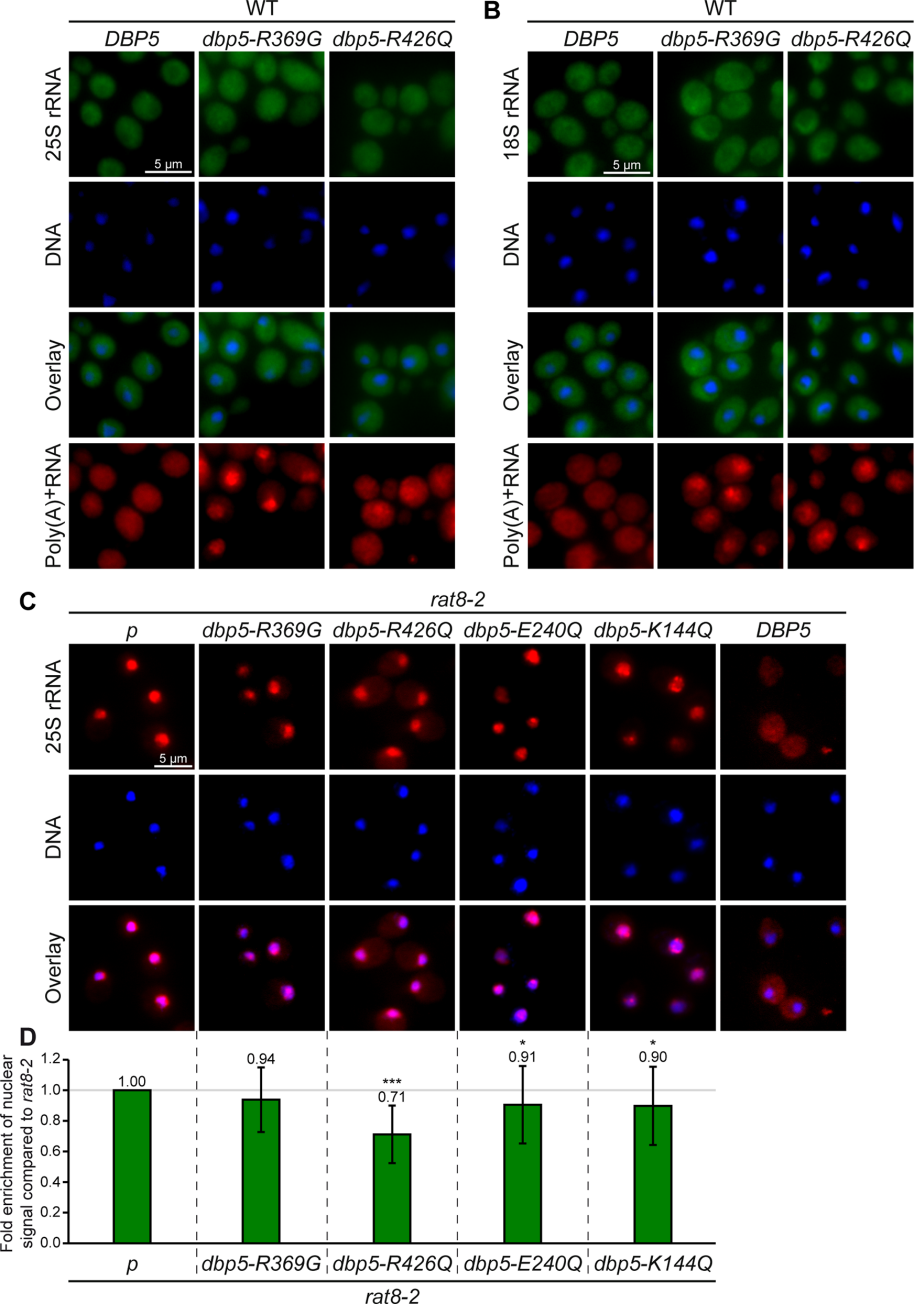
The ATPase activity of Dbp5 is dispensable for nuclear export of pre-ribosomal particles. **(A-B)** Dominant-negative ATPase-deficient *dbp5* mutants do not affect ribosomal transport. Fluorescence *in situ* hybridization experiments with wild type cells overexpressing *DBP5*, *dbp5-R369G* and *dbp5-R426Q* after 1.5 h galactose induction with probes against the 25S rRNA (A) and the 18S rRNA (B) reveal wild typical distribution of the fluorescent signals in contrast to the nuclear accumulations of the poly(A)^+^RNA. **(C)** The cytoplasmic filament-bound ATPase-deficient mutant *dbp5-R426Q* partially rescues the ribosomal transport defects of *rat8-*2. Fluorescence *in situ* hybridization experiments with Cy3-labled probes against 25S rRNAs are shown in *rat8-*2 cells with an empty vector (*p*) or overexpressing *dbp5-R369G*, *dbp5-R426Q*, *dbp5E240Q*, *dbp5K144Q* and *DBP5* after 1 h galactose induction. All cells were shifted for the last 30 min of galactose induction to 37°C. **(D)** Statistical analyses of **(C)** show the average enrichment of the nuclear signal compared to *rat8-*2 cells. The nuclear signals of at least 10 cells were set into relation with the whole cell and the signal obtained in wild type. The resulting ratios were compared to *rat8-*2. Error bars represent the standard deviation and p-values were calculated by an unpaired students t-test (*** = p<0.001 and * = p<0.05).

Localized at the cytoplasmic side of the NPC, Dbp5 was proposed to utilize its ATPase activity for displacement of export factors from emerging mRNAs [15, 17]. To investigate if the regulated ATPase cycle of Dbp5 is also needed for ribosomal transport, we analyzed the impact of ATPase-deficient mutants of *DBP5* on ribosomal export. As such mutations are lethal in yeast in the absence of the wild type protein, we overexpressed the ATPase-deficient allele *dbp5-R426Q* or the mutant *dbp5-R369G*, which has a reduced ATPase activity, in a *DBP5* wild type strain, in which the mutations cause dominant-negative effects on growth and mRNA export [41]. Indeed, overexpression of both alleles does not lead to defects in ribosomal transport, while mRNA export is clearly affected (Fig 6A and 6B) demonstrating that the expression of *dbp5-R369G* and *dbp5-R426Q* was efficiently induced. These results suggest that the ability of Dbp5 to hydrolyze ATP might not be required for efficient ribosomal transport. The dominant-negative effect on mRNA export of dbp5-R369G is caused by its competition with wild type Dbp5 for Gle1 [41], which is the ATP-hydrolysis stimulating co-factor of Dbp5 [18, 19]. Thus, Gle1 induced stimulation might not be needed for the function of Dbp5 in nuclear export of pre-ribosomal subunits.

This interpretation is further supported by analyses of different mutants of *GLE1*. While *gle1* mutants have severe defects in nuclear mRNA export (S7E Fig and [42]) emphasizing the importance of functional Gle1 in mRNA export, only few cells slightly accumulate ribosomal rRNAs (S7A and S7B Fig) or ribosomal reporter proteins (S7C and S7D Fig) in their nuclei in accordance to previously published ribosomal export screens [7, 27]. Together, these findings suggest that Gle1 is not required to stimulate the ATPase activity of Dbp5 for ribosomal transport.

To directly analyze the need of the ATPase cycle of Dbp5 for ribosomal export, we overexpressed different ATPase-deficient *dbp5* alleles in the temperature-sensitive *rat8-2* mutant and found that overexpression of *dbp5-R426Q* leads to a partial rescue of the ribosomal transport defects in these cells (Fig 6C and 6D). A nuclear accumulation of 25S rRNAs is still visible in these cells, but the average intensity of the nuclear signal compared to the whole cell is decreased to approximately 70% of that of *rat8-2* cells, while the mRNA export defect is not reduced (data not shown). The mutated protein dbp5-R426Q has a reduced RNA and ATP binding activity, but is still localized to the nuclear rim and able to bind Nup159 [41]. These findings indicate that rather the positioning of Dbp5 to the cytoplasmic side of the NPC than the ability of Dbp5 to bind RNAs and to hydrolyze ATP might be required for nuclear export of pre-ribosomal particles. In support of this interpretation, the ATPase-deficient dbp5-E240Q and dbp5-K144Q proteins are lost from NPCs and predominantly cytoplasmic [41] and their overexpression does not substantially rescue the ribosomal transport defects of *rat8-2* (Fig 6C and 6D). Interestingly, overexpressed *dbp5-R369G* does also not alter the intensity of ribosomal export defects in *rat8-2* cells, although the mutated protein is localized to the nuclear rim. In contrast to dbp5-R426Q, however, this mutant is mainly bound via Gle1 and Nup42 to the NPC [41]. Therefore, only the Nup159-bound conformation of Dbp5 might sufficiently support ribosomal export. In contrast, its ATPase activity seems to be less important, since overexpression of *dbp5-R369G*, which has a remaining ATPase activity of 60% [41], does not rescue, while NPC-localized overexpressed *dbp5-R426Q* with only ~10% ATPase activity [41] is able to partially rescue the ribosomal transport defects of *rat8-2* (Fig 6C and 6D).

### Dbp5 does not displace Mex67 from transported pre-ribosomal subunits

**Fig 7:**
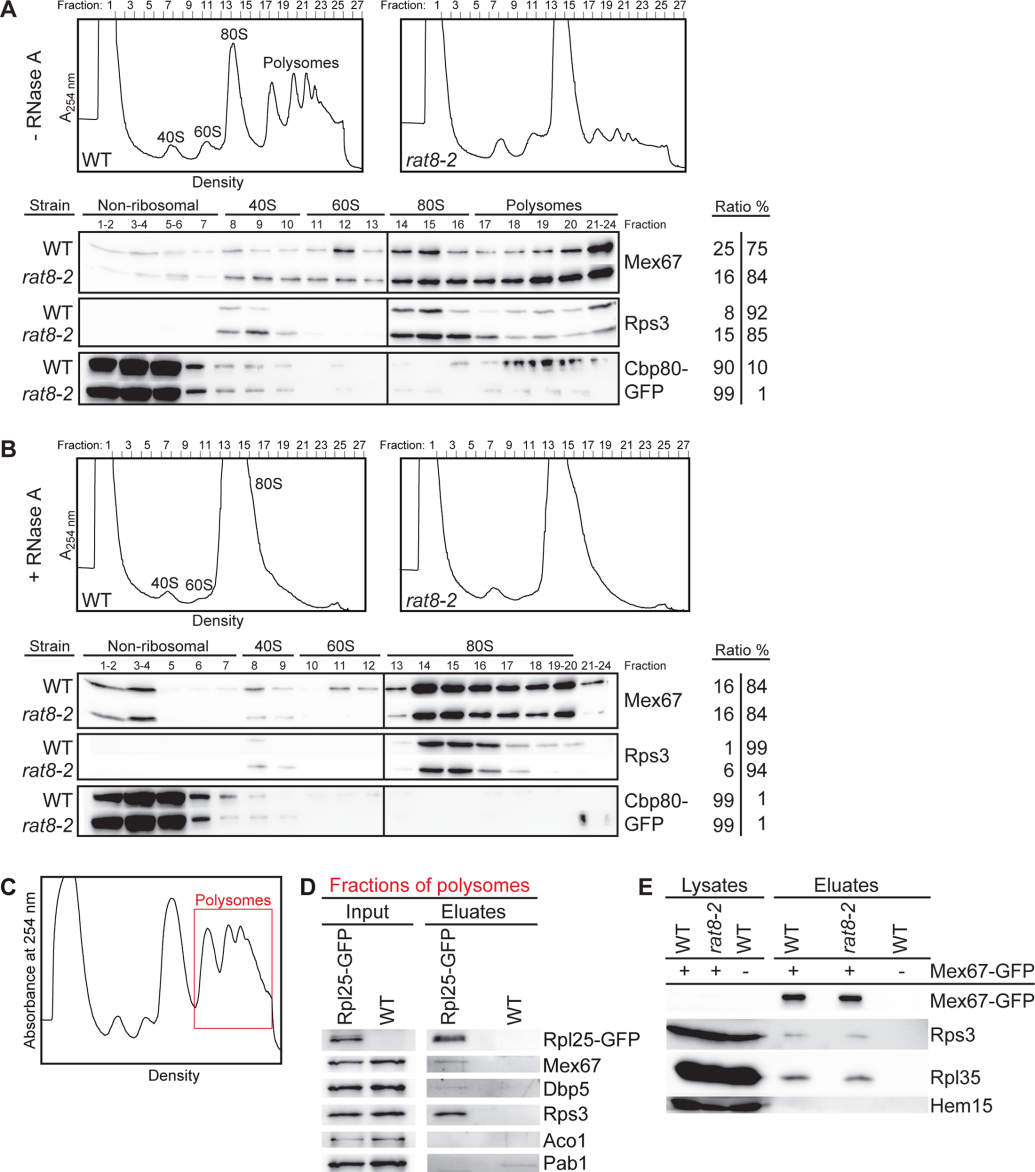
Dbp5 does not displace Mex67 from exported ribosomal particles. **(A)** Sucrose-density fractionation experiments reveal that Mex67 is associated with polysome-containing fractions from wild type and *rat8-2* cells and **(B)** remains equally ribosome bound upon RNase A treatment. The upper panel shows profiles of wild type and *rat8-2* cells upon flow through photometry (A_254nm_) from ultra-centrifuged sucrose gradients. Before loading, the cells were shifted for 1 h to 37°C and half of the resulting lysates were treated with RNase A. The bottom panel shows the corresponding separated protein fractions in Western blot analyses with direct antibodies against Mex67 and the ribosomal protein Rps3. The mRNA-binding protein GFP-Cbp80 was expressed with a galactose-inducible promoter, subsequently detected with an anti-GFP antibody to serve as a positive control. The ratios of all proteins from the first fractions (non-ribosomal+40S+60S) and the last fractions (80S+polysomal fractions) are indicated. **(C-D)** Mex67 is RNA-independently associated with polysomes in wild type cells. Western blot analyses of Rpl25-GFP immunoprecipitations from polysomal gradient fractions show co-precipitation of Mex67. Wild type yeast lysates were separated in sucrose-density gradients that were subsequently fractionated. The polysomes-containing fractions (red framed area) were subjected to co-immunoprecipitation experiments that were treated with RNase A. The correct fractions were chosen according the flow through photometry profile displayed in **(C)**. Detection of Rps3 and Dbp5 served as positive controls. Aco1 was detected as non-binding control and the mRNA-binding protein Pab1 as control for the RNase A treatment. **(E)** The association of Mex67 with ribosomal proteins is unchanged in the *rat8-2* strain. Western blot analyses of a co-immunoprecipitation experiment with Mex67-GFP and Rps3 or Rpl35 in wild type and *rat8-2* cells, which were shifted for 1 h to 37°C, are shown upon RNase A treatment. Hem15 served as a negative control.

Our results suggest that the localization of Dbp5 to the cytoplasmic filaments of the NPC is crucial for ribosomal transport, whereas its ATPase-dependent helicase activity seems to be dispensable. As a consequence, Dbp5 should not release bound transport factors from ribosomal particles. For mRNA export, in contrast, it was suggested that Dbp5 uses its regulated ATPase cycle to displace export factors such as Mex67 from the emerging mRNAs [14, 15, 17]. Mex67 is also needed for the transport of pre-40S and pre-60S subunits, but when and how the export receptor is released from ribosomal subunits is currently unknown [9, 10]. To address this question, we performed sucrose-density gradient analyses and found that Mex67 is in fact not only part of the fractions that contain the soluble proteins and the ribosomal subunits, which it does during nuclear transport, but it is also bound to polyribosomes in wild type and *dbp5* mutants (Fig 7A and S8A Fig). These findings show that Mex67 is part of translating ribosomes and is not dissociated from the ribosomal subunits by Dbp5 immediately after transport. Interestingly, the polysomal association of Mex67 increases in *rat8-2* cells (Fig 7A), despite the fact that the defects in mRNA export lead to a reduced rate of translation. This is reflected by a flat polysome profile and a corresponding reduced presence of the ribosomal protein Rps3 and the cap-binding protein Cbp80, which binds mRNAs only in the first rounds of translation [43], in polysomal fractions. This result indicates that Mex67 is not dissociated from mRNAs in the *dbp5* mutant. To analyze the ribosome-bound Mex67 pool, we eliminated the mRNA-bound Mex67 molecules through RNase A treatment. RNase A degrades single-stranded RNAs, among them mRNAs engaged in translation, which subsequently destroys the polysomes and leads to one single 80S peak (see profiles of Fig 7B). As shown in Fig 7B and S8B Fig, Mex67 is still detectable in the 80S associated fractions in *dbp5* mutants and its amount is similar to that seen in wild type cells. These findings reveal that a pool of Mex67 is in fact always ribosome-bound and not displaced by Dbp5. The mRNA-bound Cbp80 served as a positive control for the successful mRNA degradation, as it is detectable in the polysomal fractions of wild type cells (Fig 7A), but is removed from the ribosomal fractions by the RNase A treatment (Fig 7B).

To confirm these findings, we immunoprecipitated the large ribosomal protein Rpl25-GFP from polysomes-containing fractions of wild type cells and treated the samples with RNase A. As visible in Fig 7C and 7D, Mex67 can be weakly, but specifically co-precipitated with Rpl25-GFP in contrast to the Poly(A)tail-binding protein Pab1, which is not ribosome bound, but indicates that the RNase A treatment was successful. These findings indicate that Mex67 is at least transiently bound to translating ribosomes in wild type cells.

Together, these results suggest that different pools of Mex67 exist: An mRNA-bound pool that is dissociated by Dbp5 upon transport through the NPC, and a Mex67 pool that remains associated with the exported pre-ribosomal subunits until translation and is not displaced by Dbp5 at the NPC. To further support a model, in which Dbp5 does not displace Mex67 from ribosomal particles, we performed co-immunoprecipitation studies in *dbp5* mutants and found that the binding of Mex67 to the ribosomal proteins Rps3 and Rpl35 does not change in *rat8-2* cells as compared to wild type (Fig 7E). *Vice versa*, pulling the GFP-tagged large ribosomal protein Rpl11b results in similar amounts of co-precipitated Mex67 in *rat8-2* and wild type strains (S8C Fig). These results show that Dbp5 does not release Mex67 from ribosomal subunits.

### Dbp5 directly binds to the export receptor Mex67 *in vitro*

**Fig 8:**
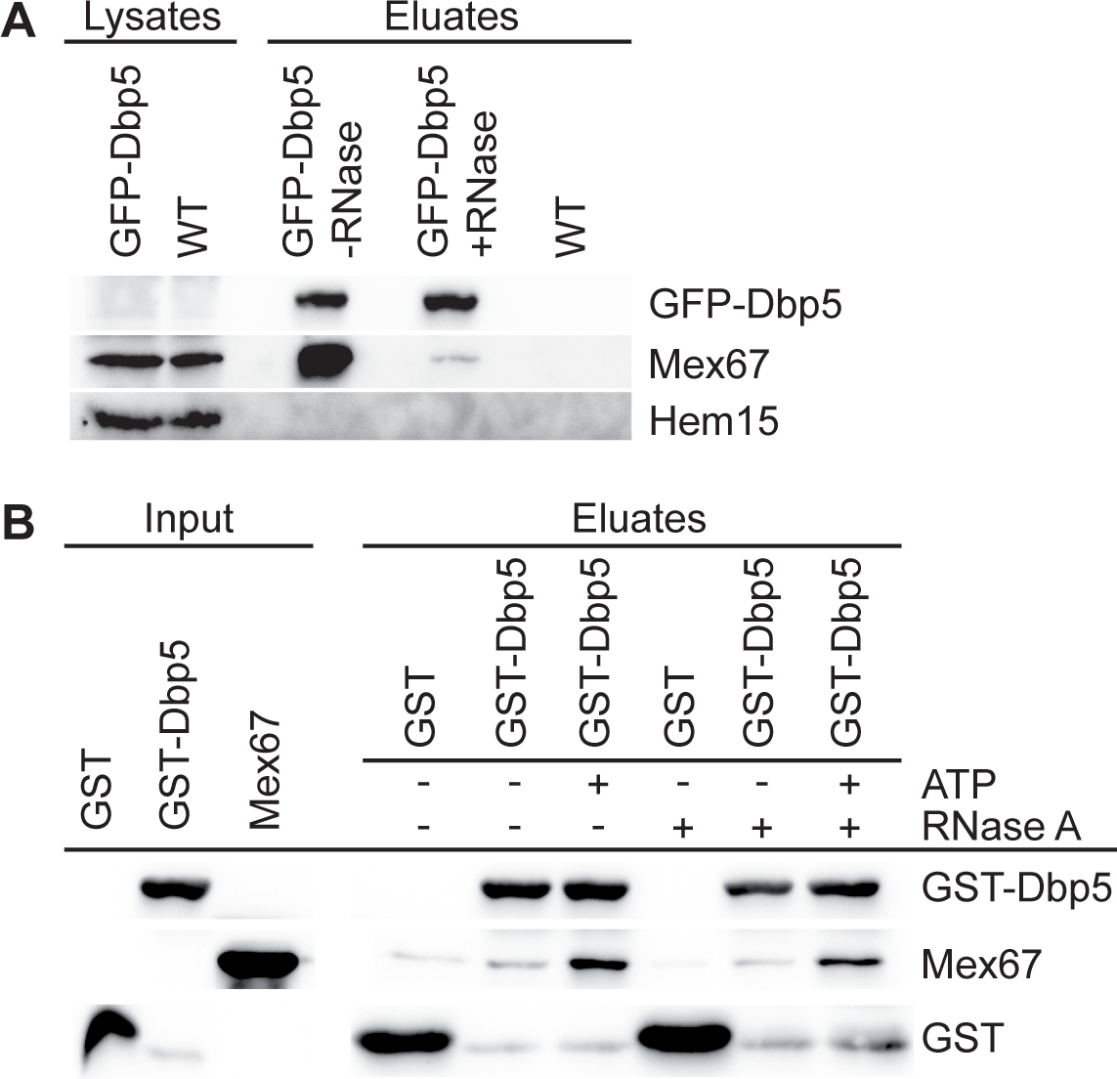
Dbp5 interacts with Mex67 *in vivo* and *in vitro*. **(A)** Dbp5 interacts mainly RNA-dependently with Mex67 *in vivo*. Western blot analyses of GFP-Dbp5 immunoprecipitations show strong co-precipitation of Mex67 without RNase A treatment, while addition of RNase A leads to a weaker, but reproducible interaction with Mex67. Detection of Hem15 served as non-binding control. **(B)** Direct interaction of recombinant GST-Dbp5 and Mex67, which is increased by ATP addition. Western blot analyses of Glutathione Sepharose pull-downs with GST-Dbp5 or GST and the purified heterodimer His-Mtr2 and Mex67 are shown in the presence or absence of 1 mM ATP and 0.2 mg/ml RNase A. The corresponding Coomassie-stained gel is depicted in S8D Fig.

Our results have uncovered differences in the transport of large RNPs. While the presence of Dbp5 at the cytoplasmic side of the NPC seems to be required for both, mRNA and ribosomal transport, its helicase activity might only be necessary for the nuclear export of mRNAs. This raises the question of how Dbp5 would facilitate ribosomal transport without utilizing its helicase activity. One possibility to support directionality of a transport event is to prevent the backsliding of the export receptor-covered transport cargo by binding the export factors and thus capturing the whole particle at the cytoplasmic side. To investigate if Dbp5 is able to bind Mex67, we analyzed the physical interaction between both proteins *in vivo* and *in vitro*. Co-immunoprecipitation studies revealed that Dbp5 and Mex67 mainly associate *in vivo* via their concomitant binding to single-stranded RNAs (Fig 8A), as reported previously [14]. However, in addition a slight RNA-independent interaction is visible (Fig 8A), which reflects a protein-protein contact. To determine whether both proteins directly interact, we performed *in vitro*-binding studies with recombinant GST-tagged Dbp5 and the purified export receptor, which is soluble only as a heterodimer, His-Mtr2+Mex67. Indeed, we found a physical interaction between these factors (Fig 8B and S8D Fig), revealing a direct contact between them. Moreover, their binding is even increased in the presence of ATP and is not influenced by the treatment with RNase A. Thus, the enhanced interaction does not depend on the increased affinity of ATP-Dbp5 for RNA [15, 19]. These results indicate that Dbp5 might preferentially bind Mex67 in a nucleotide-bound conformation, which might be the ATP- or ADP-bound state, since Dbp5 could hydrolyze the ATP. One possible model is that this contact might capture the exported pre-ribosomal subunit to the NPC exit and could prevent the back-sliding of the whole particle by covering Mex67 and possibly also other transport factors. This, in addition to the Xpo1-mediated directional transport might carry the ribosomal subunits into the cytoplasm.

Taken together, our data uncover differences in the transport mode of Dbp5 for different large RNPs and reveal a novel function for Dbp5 in ribosomal transport, in which it might not require its helicase activity.

## Discussion

Our systematic analyses of ribosomal export in different *dbp5* mutants revealed that all strains show defects in the cellular localization of ribosomal RNAs and proteins (Fig 2 and S1–S3 Figs). These findings indicate a requirement of Dbp5 for the nuclear export of both, pre-40S and pre-60S subunits. Moreover, our results suggest a direct involvement of Dbp5 in ribosomal transport that is not affected by the malfunction of mRNA export in the different *dbp5* mutants, because the intensities of the ribosomal transport defects do not correlate with the mRNA export defects (Fig 2D). Therefore, a secondary reason such as the limitation of a jointly used export factor like Mex67, which is not recycled from mRNAs in *dbp5* mutants [14], can be excluded. In such a case, a strong mRNA export defect would always result in a comparably strong ribosomal transport defect. Another concern might be that many of the mRNAs that accumulate in the nuclei of the *dbp5* mutants encode ribosomal proteins, so that their expression rate might be reduced. It seems conceivable that their subsequent lack in the nucleolus can cause ribosomal subunit mislocalizations. However, since not only rRNAs accumulate in the nuclei, but also ribosomal reporter proteins (Fig 2 and S2 Fig), it is obvious that they are visibly expressed and therefore this possible secondary reason can also be excluded. Moreover, the onset of the ribosomal export defects is fast (S3 Fig), which further argues for a primary effect.

Thus, the differences in mRNA and ribosomal export defects in the different temperature-sensitive *dbp5* mutants suggest unrelated functions for Dbp5 in both transport pathways, possibly with different interacting partners and utilization of different domains of the protein. All mutant alleles contain point mutations that cause amino acid substitutions in the helicase core (S9 Fig), which might diminish the ability to bind RNA, hydrolyze ATP or interact with the co-factors, but it is also possible that the overall protein structure is changed [13, 16]. Therefore, an entire biochemical and structural characterization would be necessary to dissect the effects of the individual mutants.

Another indirect effect that might cause the observed nuclear accumulation of pre-ribosomal particles could be fragmentation of the nucleolus into different foci that is typically visible in mRNA export mutants [29, 44]. However, we found no correlation between nucleolar fragmentations (S10A and S10B Fig) and the intensity of the ribosomal export defects in the different *dbp5* mutants (Fig 2). Furthermore, *gle1-4* cells show strong morphological changes of their nucleoli with distribution into several foci (S10A and S10B Fig and [42]), but barely failures in ribosomal transport (S7 Fig). In addition, nuclear accumulation of 25S rRNAs is still visible in *rpb1-1 rat8-2* double mutants, in which the nucleoli are less fragmented than in *rat8-2* cells (S10C Fig and [44]). These results indicate that nucleolar fragmentation is a phenotype that is typical for mRNA export mutants and cannot be the only reason for the detected ribosomal export defects in our assays.

Likewise, defects in the synthesis and maturation of ribosomal subunits as primary cause of the observed ribosomal mislocalizations are rather unlikely (Fig 3). Even though a slightly delayed 35S pre-rRNA processing is visible in the *dbp5* mutants, similar variations in the rRNA levels are detectable and were already described for the established export mutants *rat7-1* and *xpo1-1* (Fig 3A) [7, 28, 29]. Thus, such alterations in the rRNA processing in these mutants seem to be a general phenotype of nuclear RNP-export mutants. Moreover, the overall production of ribosomal subunits is not changed in the *dbp5* mutants (Fig 3B), indicating that Dbp5 is not primarily involved in the biogenesis of ribosomal subunits.

Instead, the genetic and physical interactions of Dbp5 with established ribosomal transport factors (Fig 4 and S4 Fig) rather argue for a direct role of Dbp5 in the nuclear export of pre-ribosomal subunits. While an interaction with Xpo1 might additionally occur independent from ribosomal transport [36], the physical interactions of Dbp5 with the export adapter proteins Nmd3 and Rio2 reflect its physical contact with the pre-ribosomal subunit export complexes.

However, how does Dbp5 support ribosomal transport? Similar to mRNA export, Dbp5 does not mainly shuttle with its transport cargo (Fig 5A), but rather seems to act on the pre-ribosomal subunits from the cytoplasmic filaments of the NPC right upon their appearance in the cytoplasm (Fig 5B and 5C). In fact, loss of Dbp5 from the NPC in the *nup159* mutants *rat7-1* and *rat7ΔN* lead to mild ribosomal and strong mRNA transport defects (Fig 5B and S5 Fig), as also partly reported previously [27, 29, 30, 45]. An explanation for this observation would be that rather the loss of the Dbp5 tethering to the NPC, than Nup159’s function as an ADP-release factor for Dbp5 [17] might be important for ribosomal transport. Even though Dbp5 is released from the NPC in *rat7-1* and *rat7ΔN* [36], its presence in the cytoplasm close to the pore might be sufficient to support nuclear export of pre-ribosomal subunits to some extent. Nevertheless, another possibility could be that Nup159 acts independent of Dbp5 in ribosomal transport or via a different still unknown mechanism, for example in facilitating nuclear import of Dbp5. However, such scenario seems to be unlikely, as overall protein import into the nucleus and NPC integrity is not disturbed in the *nup159* mutants [45, 46]. Interestingly, a Dbp5-independent function of Nup159 in pre-40S export was discussed earlier [29]. The authors observed defects in the transport of pre-40S subunits only in *rat7-1* mutants lacking the NPC-anchoring C-terminus, but not in *rat7ΔN* lacking the interaction domain for Dbp5 or in *rat8-*2 cells and concluded that only the C-terminus of Nup159 is needed for efficient ribosomal transport. However, they shifted the cells to the semi-permissive temperature of 34°C, which might not be sufficient to induce the ribosomal transport defects in *rat8-*2 or *rat7ΔN*. In addition, the effect on pre-60S subunit transport was not analyzed. Therefore, these studies are not sufficient to proof a Dbp5-independent function of Nup159. Our results rather argue that Nup159 might be required for the correct localization of Dbp5 for ribosomal transport. In fact, we detected ribosomal export defects in *dbp5* mutants (Fig 2 and S1–S3 Figs) and in *rat7ΔN* cells (S5B Fig). This particular mutant is directly linked to Dbp5 function, because rat7ΔN proteins specifically lack the interaction domain for Dbp5. Furthermore, overexpression of the ATPase-deficient allele *dbp5-R426Q*, which despite its defective enzymatic function is still associated with the cytoplasmic fibrils, partially rescues the ribosomal transport defects of *rat8-2*, while those alleles that release dbp5 from the NPC do not have this effect (Fig 6C and 6D). The model that Dbp5 needs to be located at the NPC for efficient nuclear export of pre-ribosomal subunits is further supported by the fact that Dbp5 is still localized to the nuclear rim in the *gle1* mutants (S11 Fig), which show no major ribosomal export defects (S7 Fig).

Acting from the cytoplasmic side of the NPC, the contact between Dbp5 and the pre-ribosomal subunits should take place during late export steps (Fig 5). Moreover, this contact is presumably quite short, because Dbp5 does not accumulate together with Nmd3 on immature pre-60S subunits (Fig 5C), which accumulate in the cytoplasm of biogenesis defective *drg1-18* cells ([39, 40] and S6 Fig). Rather, Dbp5 seems to act briefly on the pre-ribosomal subunits that emerge at the cytoplasmic side of the NPC, very similar to its function in nuclear mRNA export. However, strikingly and in contrast to mRNA transport, the ATPase-dependent remodelling activity of Dbp5 seems to be dispensable for the nuclear export of pre-ribosomal subunits (Figs 6 and 7). In mRNA export, Dbp5 was suggested to generate directionality to the transport event by releasing the export receptor Mex67 from the emerging mRNPs at the cytoplasmic side of the NPC [14]. For this remodelling activity its regulated ATPase cycle is needed [15, 17]. In contrast, our results suggest that Dbp5 does not require its ATPase activity for ribosomal transport (Fig 6 and S7 Fig) and does not displace Mex67 from the ribosomal particles (Fig 7 and S8 Fig). In fact, dominant-negative ATPase-deficient *dbp5* alleles only affect mRNA, but not ribosomal export of wild type cells (Fig 6A and 6B) and different mutants of its ATPase activity-stimulating factor Gle1 show only very faint ribosomal transport defects (S7 Fig and [7, 27]) suggesting that Gle1 and stimulation of ATP-hydrolysis of Dbp5 is not required during ribosomal transport. It might be conceivable that another ribosomal transport specific stimulating factor for Dbp5 might exist. However, this possibility seems rather unlikely, because the dominant-negative effect on mRNA export of dbp5-R369G is caused by its competition for Gle1 that leads to less ATPase stimulation of wild type Dbp5 [41]. Thus, another co-factor should also be captured by dbp5-R369G and thus influences the activity of wild type Dbp5 when overexpressed. This would consequently lead to ribosomal transport defects that are clearly not detectable (Fig 6A and 6B). Together, these results suggest that neither Gle1 nor any other, ribosomal transport specific stimulating factor is needed for the functionality of Dbp5 during ribosomal transport. Furthermore, ribosomal export defects of *rat8-*2 are partially rescued by the overexpression of the ATPase-deficient allele *dbp5-R426Q* (Fig 6C and 6D), which also has a reduced RNA and ATP binding activity [41]. This finding indicates that Dbp5 might function during ribosomal transport without binding of RNAs and hydrolysis of ATP.

Thus, we suggest a model, in which Dbp5 rather captures than remodels ribosomal subunits, possibly to support the Xpo1/RanGTPase-system mediated cargo release into the cytoplasm. In fact, in this situation, a second ATP consumption by the RNA-helicase Dbp5 seems rather unnecessary and a waste of energy. Moreover, it was shown recently that the ATPase-activity of the human Dbp5 (Ddx19) is also dispensable for the nuclear import of the transcription regulator MKL1 [44] indicating that Dbp5 does not always act as a helicase. Furthermore, ATP-hydrolysis independent functions have also been described for other DEAD-box proteins [47]. Thus, Dbp5 might support the translocation of pre-ribosomal particles simply by capturing them at the cytoplasmic side of the NPC by its direct binding to associated transport factors such as Mex67 (Fig 8) or other currently unknown export factors. For this function, the Nup159-associated ADP-bound conformation of Dbp5 might be favored, as the interaction between Mex67 and Dbp5 is increased in the presence of hydrolysable ATP (Fig 8B). Furthermore, only the Nup159-associated ATPase-deficient protein dbp5-R426Q is able to partially rescue ribosomal transport defects when overexpressed in *rat8-2* (Fig 6C and 6D). Probably, no complete rescue is possible, because a) the mutated rat8-2 protein is still present and b) the dbp5-R426Q protein has a reduced ATP-binding activity [41] and should consequently be mostly bound to Nup159 in a nucleotide-free conformation.

Our finding that Mex67 is not immediately released from the transported pre-ribosomal particles in the cytoplasm and is therefore part of actively translating ribosomes is striking. In particular, because earlier experiments detected only some Mex67 bound to polysomes [48]. The use of a specific antibody directed against Mex67 instead of using a tagged version of the export receptor and overexpression from a plasmid might have caused these discrepancies. The association of Mex67 with translating ribosomes leads to further questions such as: Does Mex67 have a function in translation, or is it simply recycled to a later time point? Nevertheless, our results uncover striking differences in the transport mechanisms of both large RNPs. But why can Dbp5 act so differently on Mex67 when it is bound to mRNAs or to ribosomal particles? The major difference between these transport pathways is that the mRNA-binding of Mex67 is mediated by adapter proteins such as Gbp2, Hrb1, Npl3, Yra1 and Nab2 [49, 50], while pre-ribosomal subunits recruit Mex67 directly through binding to the 5S rRNA [9]. The direct RNA-binding might require other mechanisms for dissociation. As it was shown before that Dbp5 also displaces Nab2 from the mRNA [15], Mex67 might be released together with its adapter protein. Whether Dbp5 is needed to dissociate other currently known transport factors from the arriving ribosomal particles seems rather unlikely, because the helicase-activity of Dbp5 seems to be generally dispensable for its function in ribosomal transport (Fig 6 and S7 Fig).

In summary, our data suggest that even though Dbp5 is required for both RNP transport processes, it has different ways of supporting them (S12 Fig). In mRNA export, it uses its ATPase activity to displace Mex67 and to generate directionality, while in ribosomal transport its function as a helicase seems to be not required. One possible model is that Dbp5 might support the nuclear export of pre-ribosomal subunits simply by shielding bound transport factors such as Mex67 and thereby capturing the ribosomal particles at the cytoplasmic side of the NPC. In this way, Dbp5 could enhance the Xpo1- and RanGTPase-driven directional transport process. In any case, Dbp5 is required at the cytoplasmic side of the NPC for both large RNP transport pathways–the mRNA and the ribosomal nuclear export pathways–and it acts with different mechanisms on both transport cargos leaving Mex67 attached to ribosomal subunits.

## Supporting Information

S1 Fig Split channels for the FITC and the Hoechst signals and their overlays of Fig 1B and 1C. (TIF)

S2 Fig Reporter proteins of the 60S and the 40S ribosomal subunit accumulate in the nuclei of *dbp5* mutants. Fluorescence microscopy images of Rpl25-GFP **(A),** Rps2-GFP **(B)** and Nmd3-GFP **(C)** are shown in *xpo1-1*, *rat8-1*, *rat8-2*, *rat8-3* and *rat8-7* cells shifted for 1 h to their restrictive temperatures.
(TIF)

S3 Fig The onset of the nuclear ribosomal accumulation in *dbp5* mutants is rapid. Fluorescence *in situ* hybridization experiments with probes against the 25S **(A)** and 18S **(B)** rRNA (green) are shown together with the stained DNA (blue) in *rat8-2* and *rat8-3* cells shifted for 15 or 30 min to 37°C.
(TIF)

S4 Fig Dbp5 physically interacts with ribosomal transport factors. Western blot analyses of Xpo1-GFP and Nmd3-GFP immunoprecipitations show co-precipitation of Dbp5 **(A)** and co-precipitation of Rio2 is visible in the GFP-Dbp5 pull down **(B)**. All samples were treated with RNase A and detection of Hem15 served as non-binding control.
(TIF)

S5 Fig *Rat7/nup159* mutants show mild nuclear accumulation of pre-ribosomal particles. **(A)** Split channels for the FITC and the Hoechst signals and their overlays of Fig 5B. **(B)** Ribosomal export defects are visible in *rat7ΔN* cells lacking the interaction domain for Dbp5, but less strong than in *rat8-2* cells. Fluorescence microscopy images of *in situ* hybridization experiments with Cy3-labeled probes against the 25S rRNA are displayed for WT, *rat7ΔN* and *rat8-2* cells upon shift for 1 h to 37°C.
(TIF)

S6 Fig Immature pre-60S subunits accumulate in the cytoplasm of *drg1-18* cells. Western blot analyses of Arx1-GFP immunoprecipitations show an increased co-precipitation of Mex67 and Nmd3-myc in *drg1-18* compared to wild type cells upon temperature shift for 1 h to 37°C. Detection of Hem15 served as negative control.
(TIF)

S7 Fig Mutants of *gle1* show only marginal ribosomal transport defects. **(A-B)** Fluorescence *in situ* hybridization experiments with *gle1-4* and wild type cells upon temperature shifts for 1 h to 37°C with probes against the 25S rRNA (A) and the 18S rRNA (B) are shown. **(C-D)** Fluorescence microscopy images of the ribosomal reporter proteins Rpl25-GFP (C) and Rps2-GFP (D) are shown in *gle1-2*, *gle1-4* and wild type cells shifted for 1 h to 37°C. **(E)** Poly(A)^+^RNA accumulates in the nuclei of the different *gle1* mutants. *In situ* hybridization experiments with Cy3-labeled oligo(dT)_50_ probes are shown in *gle1-2*, *gle1-4* and wild type cells upon 1 h shift to 37°C.
(TIF)

S8 Fig Mutations of *DBP5* (*rat8-2* shown in Fig 7 and *rat8-7* in S8 Fig) does not affect the ribosomal association of Mex67. **(A-B)** Mex67 is associated with ribosomal fractions of sucrose-density gradients from wild type and *rat8-7* cells without **(A)** and with **(B)** the addition of RNase A. Upper panels show flow through photometry (A_254nm_) profiles of wild type and *rat8-7* cells shifted for 1 h to 16°C. Bottom panels show the corresponding separated protein fractions in Western blot analyses with direct antibodies against Mex67 and the ribosomal protein Rps3. **(C)** The interaction between the large ribosomal protein Rpl11b and Mex67 is not altered in the *rat8-7* strain. Western blot analyses show co-precipitated Mex67 and as a positive control Rps3 in the Rpl11b-GFP immunoprecipitation after RNase A treatment. The protein level of Mex67 is not changed in *rat8-7* cells compared to wild type upon 1 h temperature shifts to 16°C. Aco1 served as a non-binding control. **(D)** Dbp5 and Mex67 directly interact with each other. The Coomassie stained gel with the same samples of Fig 8B shows successful pull-down of GST-Dbp5 and GST with similar efficiency. The protein sizes are indicated in kDa.
(TIF)

S9 Fig Domain structure of yeast Dbp5 and positions of the amino acid substitutions in the different *dbp5/rat8* mutants. The scheme shows the 13 conserved sequence motifs that bind RNA (green), bind and hydrolyze ATP (red) or are necessary for both (blue). The positions of the amino acid substitutions in the different temperature-sensitive (at the top) and ATPase-deficient (at the bottom) *dbp5*/*rat8* mutants are indicated in yellow. The co-factors Gle1, IP_6_ and Nup159 interact with the protein surface and important interaction sites are marked in gray. Picture modified from [16].
(TIF)

S10 Fig Nucleolar fragmentation of mRNA export mutants does not correlate with the intensity of the detected ribosomal export defects (compare with Fig 2 and S7 Fig). **(A)** Fluorescence microscopy images of immunofluorescence experiments to stain Nop1 as nucleolar marker show the intensity of nulceolar fragmentation in *gle1-4*, *dbp5* mutants and wild type cells upon 1 h shift to their non-permissive temperatures. **(B)** Quantification of **(A)** displays the percentage of cells with nucleolar fragmentation. **(C)** The double mutant *rpb1-1 rat8-2* shows less nucleolar fragmentation than *rat8-2*, but still nuclear accumulation of 25S rRNAs. Fluorescence microscopy images of combined *in situ* hybridization with Cy3-labeled 25S probes and immunofluorescence against the nucleolar protein Nop1 are shown in *rpb1-1 rat8-2*, *rat8-2* and wild type cells upon shifts for 1 h to 37°C.
(TIF)

S11 Fig The nuclear rim localization of Dbp5 is not disturbed in strains mutated for *GLE1*. Fluorescence microscopy images of GFP-Dbp5 in living yeast cells are shown in *gle1-2*, *gle1-4* and wild type cells upon 1 h shift to 37°C.
(TIF)

S12 Fig Model showing the differences in the Dbp5 mediated nuclear export of mRNAs and pre-ribosomal subunits. **(A)** Model for the role of Dbp5 in mRNA transport from the nucleus into the cytoplasm. The ATPase cycle of Dbp5 and its regulation by its cofactors is necessary to displace export factors such as Mex67 and Nab2 from the mRNA and to provide directionality. **(B)** Model for the function of Dbp5 in the nuclear export of both pre-ribosomal subunits. Dbp5 does not release Mex67 from the emerging ribosomal particles and the ATPase cycle of Dbp5 is dispensable for ribosomal transport. However, the presence of Dbp5 at the cytoplasmic side of the NPC is required, possibly to capture the export factor-bound pre-ribosomal particles and prevent their backsliding into the NPC. In this way, Dbp5 could support other export factors such as Xpo1 that generate directionality to the transport process by their RanGTP-hydrolysis induced dissociation.
(TIF)

S1 Table *Saccharomyces cerevisiae* strains used in this study. (PDF)

S2 Table Plasmids used in this study. (PDF)

S3 Table Oligonucleotides used in this study. (PDF)

## References

[1] Gerhardy S, Menet AM, Pena C, Petkowski JJ, Panse VG. Assembly and nuclear export of pre-ribosomal particles in budding yeast. Chromosoma. 2014 Epub 2014/05/13. 10.1007/s00412-014-0463-z . 24817020

[2] Ho JH, Kallstrom G, Johnson AW. Nmd3p is a Crm1p-dependent adapter protein for nuclear export of the large ribosomal subunit. J Cell Biol. 2000;151(5):1057–66. Epub 2000/11/22. 11086007 10.1083/jcb.151.5.1057 PMC2174350

[3] Gadal O, Strauss D, Kessl J, Trumpower B, Tollervey D, Hurt E. Nuclear export of 60s ribosomal subunits depends on Xpo1p and requires a nuclear export sequence-containing factor, Nmd3p, that associates with the large subunit protein Rpl10p. Mol Cell Biol. 2001;21(10):3405–15. Epub 2001/04/21. 10.1128/MCB.21.10.3405-3415.2001 11313466 PMC100262

[4] Trotta CR, Lund E, Kahan L, Johnson AW, Dahlberg JE. Coordinated nuclear export of 60S ribosomal subunits and NMD3 in vertebrates. EMBO J. 2003;22(11):2841–51. Epub 2003/05/30. 10.1093/emboj/cdg249 12773398 PMC156746

[5] Thomas F, Kutay U. Biogenesis and nuclear export of ribosomal subunits in higher eukaryotes depend on the CRM1 export pathway. J Cell Sci. 2003;116(Pt 12):2409–19. Epub 2003/05/02. 10.1242/jcs.00464 jcs.00464 [pii]. . 12724356

[6] Wente SR, Rout MP. The nuclear pore complex and nuclear transport. Cold Spring Harb Perspect Biol. 2010;2(10):a000562 Epub 2010/07/16. 10.1101/cshperspect.a000562 20630994 PMC2944363

[7] Moy TI, Silver PA. Nuclear export of the small ribosomal subunit requires the ran-GTPase cycle and certain nucleoporins. Genes Dev. 1999;13(16):2118–33. Epub 1999/08/31. 10465789 10.1101/gad.13.16.2118 PMC316945

[8] Zemp I, Wild T, O'Donohue MF, Wandrey F, Widmann B, Gleizes PE, et al Distinct cytoplasmic maturation steps of 40S ribosomal subunit precursors require hRio2. J Cell Biol. 2009;185(7):1167–80. Epub 2009/07/01. 10.1083/jcb.200904048 19564402 PMC2712965

[9] Yao W, Roser D, Kohler A, Bradatsch B, Bassler J, Hurt E. Nuclear export of ribosomal 60S subunits by the general mRNA export receptor Mex67-Mtr2. Mol Cell. 2007;26(1):51–62. Epub 2007/04/17. doi: S1097-2765(07)00118-9 [pii] 10.1016/j.molcel.2007.02.018 . 17434126

[10] Faza MB, Chang Y, Occhipinti L, Kemmler S, Panse VG. Role of Mex67-Mtr2 in the nuclear export of 40S pre-ribosomes. PLoS Genet. 2012;8(8):e1002915 Epub 2012/09/08. 10.1371/journal.pgen.1002915 PGENETICS-D-12-00596 [pii]. 22956913 PMC3431309

[11] Panse VG, Johnson AW. Maturation of eukaryotic ribosomes: acquisition of functionality. Trends Biochem Sci. 2010;35(5):260–6. Epub 2010/02/09. 10.1016/j.tibs.2010.01.001 20137954 PMC2866757

[12] Tseng SS, Weaver PL, Liu Y, Hitomi M, Tartakoff AM, Chang TH. Dbp5p, a cytosolic RNA helicase, is required for poly(A)+ RNA export. EMBO J. 1998;17(9):2651–62. Epub 1998/06/20. 10.1093/emboj/17.9.2651 9564047 PMC1170606

[13] Snay-Hodge CA, Colot HV, Goldstein AL, Cole CN. Dbp5p/Rat8p is a yeast nuclear pore-associated DEAD-box protein essential for RNA export. EMBO J. 1998;17(9):2663–76. Epub 1998/06/20. 10.1093/emboj/17.9.2663 9564048 PMC1170607

[14] Lund MK, Guthrie C. The DEAD-box protein Dbp5p is required to dissociate Mex67p from exported mRNPs at the nuclear rim. Mol Cell. 2005;20(4):645–51. Epub 2005/11/26. doi: S1097-2765(05)01675-8 [pii] 10.1016/j.molcel.2005.10.005 . 16307927

[15] Tran EJ, Zhou Y, Corbett AH, Wente SR. The DEAD-box protein Dbp5 controls mRNA export by triggering specific RNA:protein remodeling events. Mol Cell. 2007;28(5):850–9. Epub 2007/12/18. doi: S1097-2765(07)00631-4 [pii] 10.1016/j.molcel.2007.09.019 . 18082609

[16] Tieg B, Krebber H. Dbp5—from nuclear export to translation. Biochim Biophys Acta. 2013;1829(8):791–8. Epub 2012/11/07. 10.1016/j.bbagrm.2012.10.010 . 23128325

[17] Noble KN, Tran EJ, Alcazar-Roman AR, Hodge CA, Cole CN, Wente SR. The Dbp5 cycle at the nuclear pore complex during mRNA export II: nucleotide cycling and mRNP remodeling by Dbp5 are controlled by Nup159 and Gle1. Genes Dev. 2011;25(10):1065–77. Epub 2011/05/18. doi: 25/10/1065 [pii] 10.1101/gad.2040611 21576266 PMC3093122

[18] Alcazar-Roman AR, Tran EJ, Guo S, Wente SR. Inositol hexakisphosphate and Gle1 activate the DEAD-box protein Dbp5 for nuclear mRNA export. Nat Cell Biol. 2006;8(7):711–6. Epub 2006/06/20. doi: ncb1427 [pii] 10.1038/ncb1427 . 16783363

[19] Weirich CS, Erzberger JP, Flick JS, Berger JM, Thorner J, Weis K. Activation of the DExD/H-box protein Dbp5 by the nuclear-pore protein Gle1 and its coactivator InsP6 is required for mRNA export. Nat Cell Biol. 2006;8(7):668–76. Epub 2006/06/20. doi: ncb1424 [pii] 10.1038/ncb1424 . 16783364

[20] Gross T, Siepmann A, Sturm D, Windgassen M, Scarcelli JJ, Seedorf M, et al The DEAD-box RNA helicase Dbp5 functions in translation termination. Science. 2007;315(5812):646–9. Epub 2007/02/03. doi: 315/5812/646 [pii] 10.1126/science.1134641 . 17272721

[21] Bolger TA, Folkmann AW, Tran EJ, Wente SR. The mRNA export factor Gle1 and inositol hexakisphosphate regulate distinct stages of translation. Cell. 2008;134(4):624–33. Epub 2008/08/30. doi: S0092-8674(08)00779-4 [pii] 10.1016/j.cell.2008.06.027 18724935 PMC2601711

[22] Lei EP, Krebber H, Silver PA. Messenger RNAs are recruited for nuclear export during transcription. Genes Dev. 2001;15(14):1771–82. Epub 2001/07/19. 10.1101/gad.892401 11459827 PMC312744

[23] Baierlein C, Hackmann A, Gross T, Henker L, Hinz F, Krebber H. Monosome formation during translation initiation requires the serine/arginine-rich protein Npl3. Mol Cell Biol. 2013;33(24):4811–23. Epub 2013/10/09. 10.1128/MCB.00873-13 MCB.00873-13 [pii]. 24100011 PMC3889561

[24] Hackmann A, Gross T, Baierlein C, Krebber H. The mRNA export factor Npl3 mediates the nuclear export of large ribosomal subunits. EMBO Rep. 2011;12(10):1024–31. Epub 2011/08/20. 10.1038/embor.2011.155 embor2011155 [pii]. 21852791 PMC3185341

[25] Wu H, Becker D, Krebber H. Telomerase RNA TLC1 shuttling to the cytoplasm requires mRNA export factors and is important for telomere maintenance. Cell Rep. 2014;8(6):1630–8. Epub 2014/09/16. 10.1016/j.celrep.2014.08.021 . 25220466

[26] Milkereit P, Strauss D, Bassler J, Gadal O, Kuhn H, Schutz S, et al A Noc complex specifically involved in the formation and nuclear export of ribosomal 40 S subunits. J Biol Chem. 2003;278(6):4072–81. Epub 2002/11/26. 10.1074/jbc.M208898200 . 12446671

[27] Stage-Zimmermann T, Schmidt U, Silver PA. Factors affecting nuclear export of the 60S ribosomal subunit in vivo. Mol Biol Cell. 2000;11(11):3777–89. Epub 2000/11/10. 11071906 10.1091/mbc.11.11.3777 PMC15036

[28] Del Priore V, Snay CA, Bahr A, Cole CN. The product of the Saccharomyces cerevisiae RSS1 gene, identified as a high-copy suppressor of the rat7-1 temperature-sensitive allele of the RAT7/NUP159 nucleoporin, is required for efficient mRNA export. Mol Biol Cell. 1996;7(10):1601–21. Epub 1996/10/01. 8898365 10.1091/mbc.7.10.1601 PMC276009

[29] Gleizes PE, Noaillac-Depeyre J, Leger-Silvestre I, Teulieres F, Dauxois JY, Pommet D, et al Ultrastructural localization of rRNA shows defective nuclear export of preribosomes in mutants of the Nup82p complex. J Cell Biol. 2001;155(6):923–36. Epub 2001/12/12. 10.1083/jcb.200108142 jcb.200108142 [pii]. 11739405 PMC2150900

[30] Moy TI, Silver PA. Requirements for the nuclear export of the small ribosomal subunit. J Cell Sci. 2002;115(Pt 14):2985–95. Epub 2002/06/26. . 12082158 10.1242/jcs.115.14.2985

[31] Hedges J, West M, Johnson AW. Release of the export adapter, Nmd3p, from the 60S ribosomal subunit requires Rpl10p and the cytoplasmic GTPase Lsg1p. EMBO J. 2005;24(3):567–79. Epub 2005/01/22. doi: 7600547 [pii] 10.1038/sj.emboj.7600547 15660131 PMC548654

[32] Bassler J, Grandi P, Gadal O, Lessmann T, Petfalski E, Tollervey D, et al Identification of a 60S preribosomal particle that is closely linked to nuclear export. Mol Cell. 2001;8(3):517–29. Epub 2001/10/05. doi: S1097-2765(01)00342-2 [pii]. . 11583615 10.1016/s1097-2765(01)00342-2

[33] Scarcelli JJ, Viggiano S, Hodge CA, Heath CV, Amberg DC, Cole CN. Synthetic genetic array analysis in Saccharomyces cerevisiae provides evidence for an interaction between RAT8/DBP5 and genes encoding P-body components. Genetics. 2008;179(4):1945–55. Epub 2008/08/12. doi: genetics.108.091256 [pii] 10.1534/genetics.108.091256 18689878 PMC2516071

[34] Estruch F, Cole CN. An early function during transcription for the yeast mRNA export factor Dbp5p/Rat8p suggested by its genetic and physical interactions with transcription factor IIH components. Mol Biol Cell. 2003;14(4):1664–76. Epub 2003/04/11. 10.1091/mbc.E02-09-0602 12686617 PMC153130

[35] Estruch F, Hodge C, Gomez-Navarro N, Peiro-Chova L, Heath CV, Cole CN. Insights into mRNP biogenesis provided by new genetic interactions among export and transcription factors. BMC genetics. 2012;13:80 Epub 2012/09/12. 10.1186/1471-2156-13-80 22963203 PMC3506551

[36] Hodge CA, Colot HV, Stafford P, Cole CN. Rat8p/Dbp5p is a shuttling transport factor that interacts with Rat7p/Nup159p and Gle1p and suppresses the mRNA export defect of xpo1-1 cells. EMBO J. 1999;18(20):5778–88. Epub 1999/10/16. 10.1093/emboj/18.20.5778 10523319 PMC1171644

[37] Schmitt C, von Kobbe C, Bachi A, Pante N, Rodrigues JP, Boscheron C, et al Dbp5, a DEAD-box protein required for mRNA export, is recruited to the cytoplasmic fibrils of nuclear pore complex via a conserved interaction with CAN/Nup159p. EMBO J. 1999;18(15):4332–47. Epub 1999/08/03. 10.1093/emboj/18.15.4332 10428971 PMC1171509

[38] Weirich CS, Erzberger JP, Berger JM, Weis K. The N-terminal domain of Nup159 forms a beta-propeller that functions in mRNA export by tethering the helicase Dbp5 to the nuclear pore. Mol Cell. 2004;16(5):749–60. Epub 2004/12/03. doi: S1097276504006525 [pii] 10.1016/j.molcel.2004.10.032 . 15574330

[39] Pertschy B, Saveanu C, Zisser G, Lebreton A, Tengg M, Jacquier A, et al Cytoplasmic recycling of 60S preribosomal factors depends on the AAA protein Drg1. Mol Cell Biol. 2007;27(19):6581–92. Epub 2007/07/25. 10.1128/MCB.00668-07 17646390 PMC2099225

[40] Kappel L, Loibl M, Zisser G, Klein I, Fruhmann G, Gruber C, et al Rlp24 activates the AAA-ATPase Drg1 to initiate cytoplasmic pre-60S maturation. J Cell Biol. 2012;199(5):771–82. Epub 2012/11/28. 10.1083/jcb.201205021 23185031 PMC3514788

[41] Hodge CA, Tran EJ, Noble KN, Alcazar-Roman AR, Ben-Yishay R, Scarcelli JJ, et al The Dbp5 cycle at the nuclear pore complex during mRNA export I: dbp5 mutants with defects in RNA binding and ATP hydrolysis define key steps for Nup159 and Gle1. Genes Dev. 2011;25(10):1052–64. Epub 2011/05/18. doi: 25/10/1052 [pii] 10.1101/gad.2041611 21576265 PMC3093121

[42] Murphy R, Wente SR. An RNA-export mediator with an essential nuclear export signal. Nature. 1996;383(6598):357–60. Epub 1996/09/26. 10.1038/383357a0 . 8848052

[43] Garre E, Romero-Santacreu L, De Clercq N, Blasco-Angulo N, Sunnerhagen P, Alepuz P. Yeast mRNA cap-binding protein Cbc1/Sto1 is necessary for the rapid reprogramming of translation after hyperosmotic shock. Mol Biol Cell. 2012;23(1):137–50. Epub 2011/11/11. 10.1091/mbc.E11-05-0419 22072789 PMC3248893

[44] Kadowaki T, Hitomi M, Chen S, Tartakoff AM. Nuclear mRNA accumulation causes nucleolar fragmentation in yeast mtr2 mutant. Mol Biol Cell. 1994;5(11):1253–63. Epub 1994/11/01. 7865887 10.1091/mbc.5.11.1253 PMC301150

[45] Gorsch LC, Dockendorff TC, Cole CN. A conditional allele of the novel repeat-containing yeast nucleoporin RAT7/NUP159 causes both rapid cessation of mRNA export and reversible clustering of nuclear pore complexes. J Cell Biol. 1995;129(4):939–55. Epub 1995/05/01. 7744966 10.1083/jcb.129.4.939 PMC2120496

[46] Del Priore V, Heath C, Snay C, MacMillan A, Gorsch L, Dagher S, et al A structure/function analysis of Rat7p/Nup159p, an essential nucleoporin of Saccharomyces cerevisiae. J Cell Sci. 1997;110 (Pt 23):2987–99. Epub 1998/02/07. . 9359887 10.1242/jcs.110.23.2987

[47] Linder P, Jankowsky E. From unwinding to clamping—the DEAD box RNA helicase family. Nat Rev Mol Cell Biol. 2011;12(8):505–16. Epub 2011/07/23. 10.1038/nrm3154 nrm3154 [pii]. . 21779027

[48] Windgassen M, Sturm D, Cajigas IJ, Gonzalez CI, Seedorf M, Bastians H, et al Yeast shuttling SR proteins Npl3p, Gbp2p, and Hrb1p are part of the translating mRNPs, and Npl3p can function as a translational repressor. Mol Cell Biol. 2004;24(23):10479–91. Epub 2004/11/16. doi: 24/23/10479 [pii] 10.1128/MCB.24.23.10479-10491.2004 15542855 PMC529038

[49] Hackmann A, Wu H, Schneider UM, Meyer K, Jung K, Krebber H. Quality control of spliced mRNAs requires the shuttling SR proteins Gbp2 and Hrb1. Nat Commun. 2014;5:3123 Epub 2014/01/24. 10.1038/ncomms4123 . 24452287

[50] Kelly SM, Corbett AH. Messenger RNA export from the nucleus: a series of molecular wardrobe changes. Traffic. 2009;10(9):1199–208. Epub 2009/06/26. 10.1111/j.1600-0854.2009.00944.x 19552647 PMC3702165

