# Supplementary figures and images for: Nuclear Export of Pre-Ribosomal Subunits Requires Dbp5, but Not as an RNA-Helicase as for mRNA Export
Source: PLoS One. 2016 Feb 12;11(2):e0149571. doi: 10.1371/journal.pone.0149571 (PMC4752221; doi:10.1371/journal.pone.0149571)

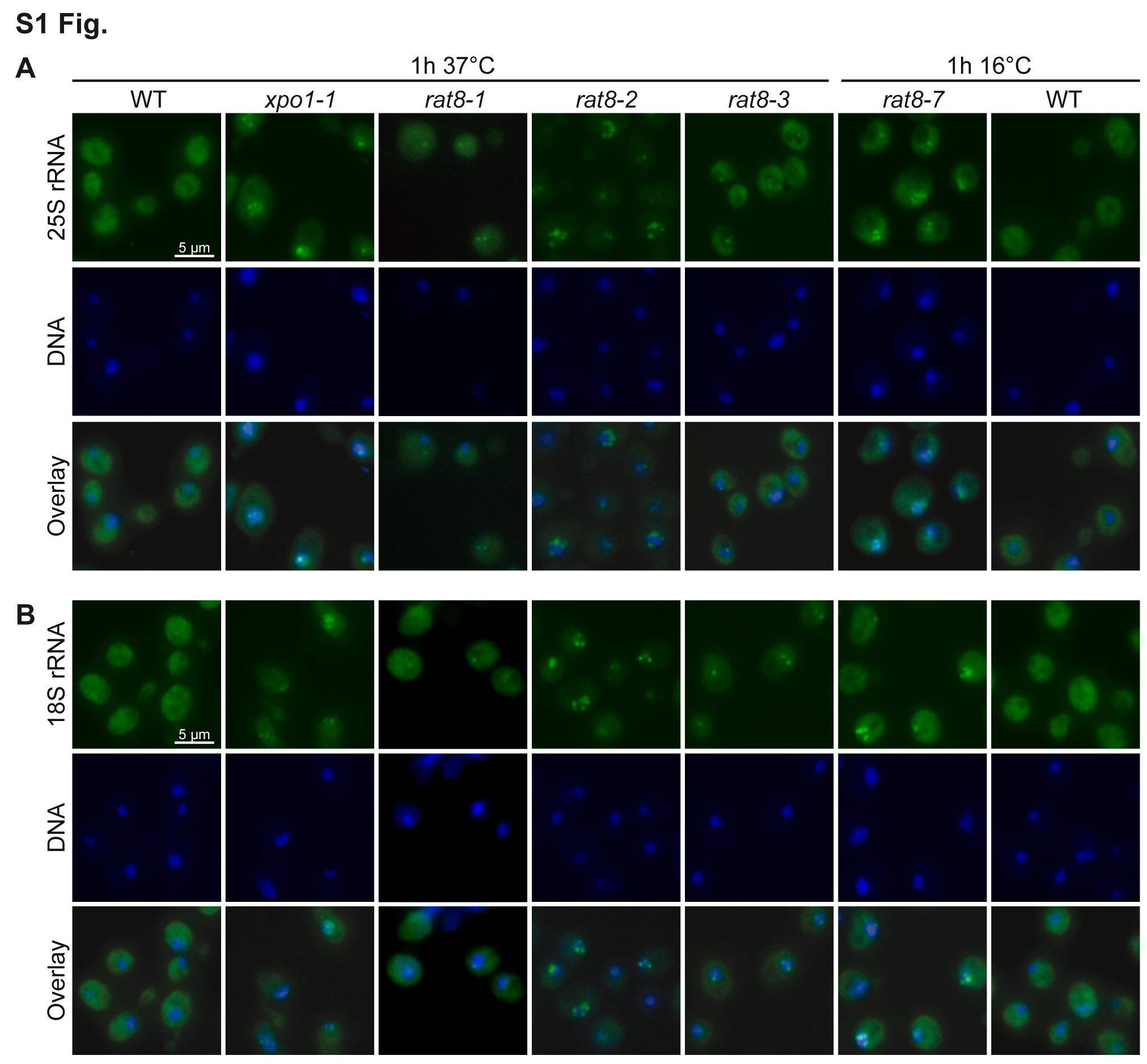

Supplement: S1 Fig — (TIF) [file pone.0149571.s001.tif]

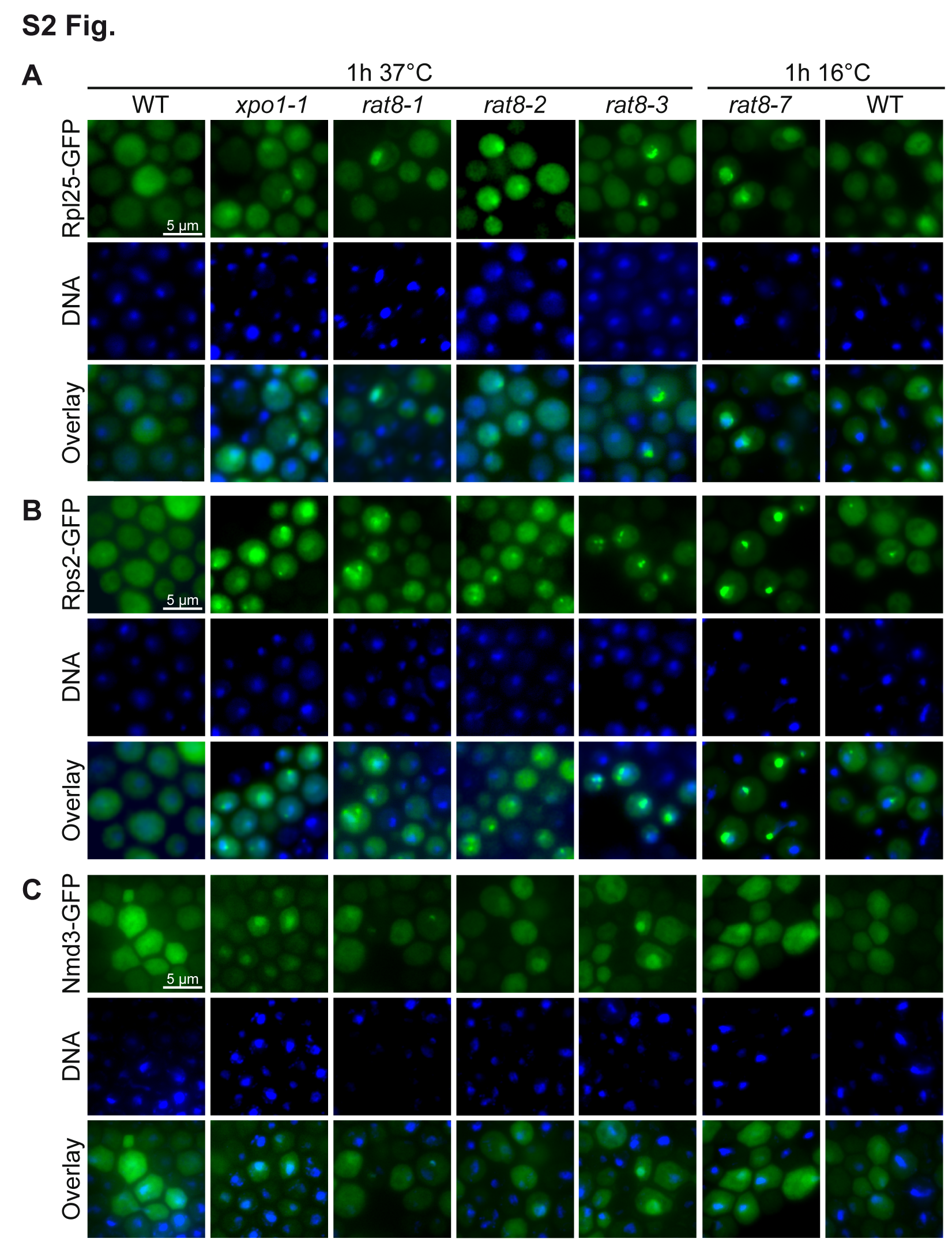

Supplement: S2 Fig — Fluorescence microscopy images of Rpl25-GFP (A), Rps2-GFP (B) and Nmd3-GFP (C) are shown in xpo1-1, rat8-1, rat8-2, rat8-3 and rat8-7 cells shifted for 1 h to their restrictive temperatures. (TIF) [file pone.0149571.s002.tif]

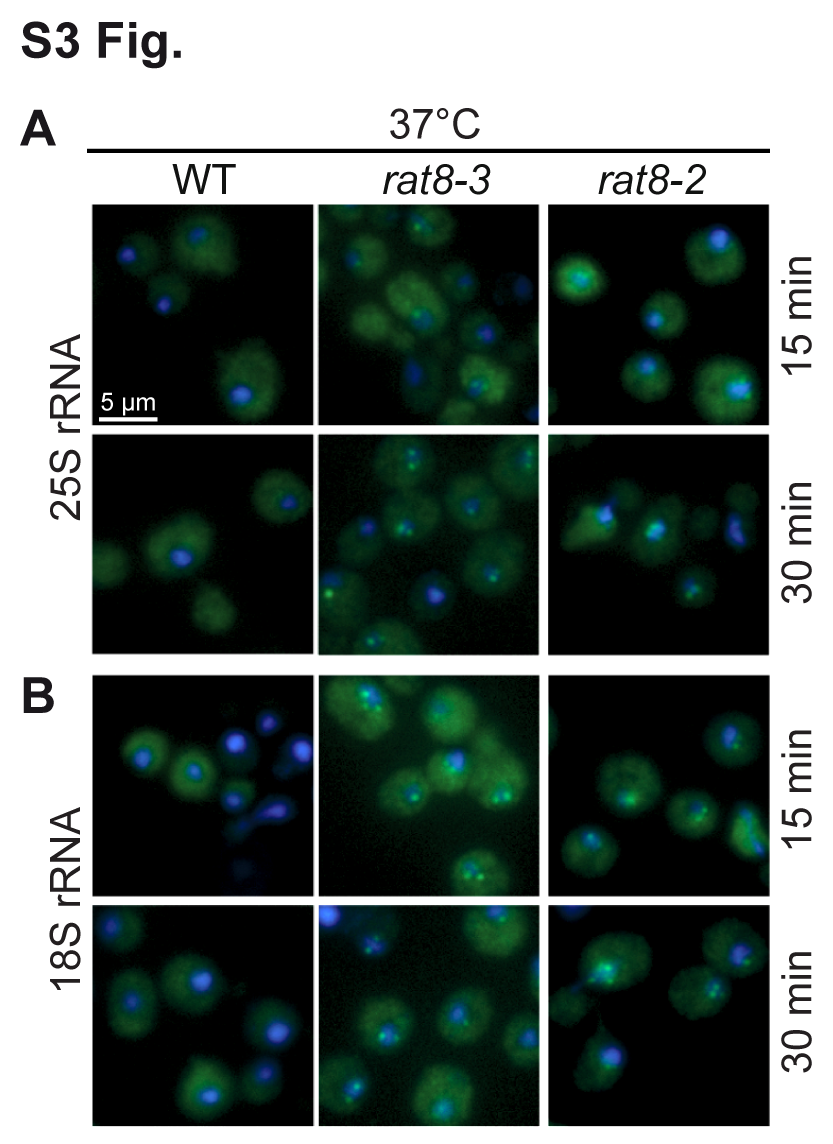

Supplement: S3 Fig — Fluorescence in situ hybridization experiments with probes against the 25S (A) and 18S (B) rRNA (green) are shown together with the stained DNA (blue) in rat8-2 and rat8-3 cells shifted for 15 or 30 min to 37°C. (TIF) [file pone.0149571.s003.tif]

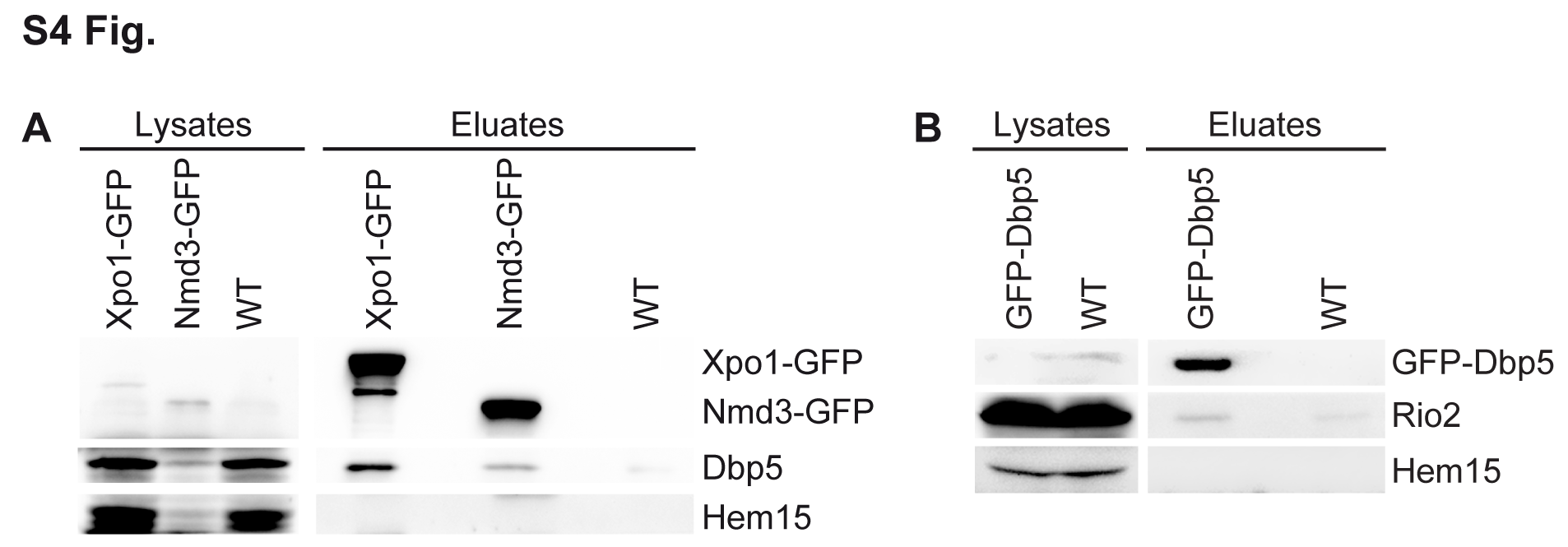

Supplement: S4 Fig — Western blot analyses of Xpo1-GFP and Nmd3-GFP immunoprecipitations show co-precipitation of Dbp5 (A) and co-precipitation of Rio2 is visible in the GFP-Dbp5 pull down (B). All samples were treated with RNase A and detection of Hem15 served as non-binding control. (TIF) [file pone.0149571.s004.tif]

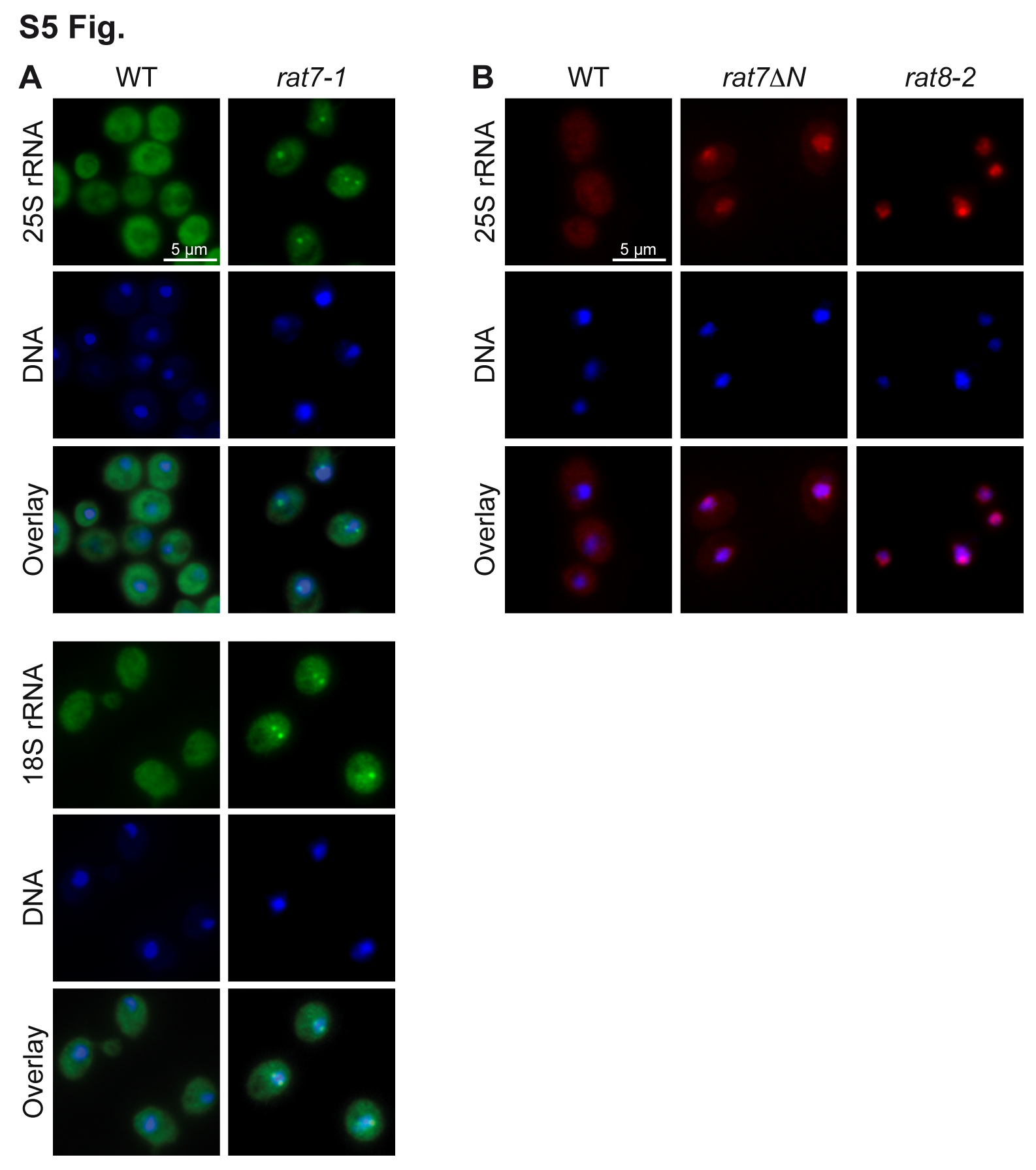

Supplement: S5 Fig — (A) Split channels for the FITC and the Hoechst signals and their overlays of Fig 5B. (B) Ribosomal export defects are visible in rat7ΔN cells lacking the interaction domain for Dbp5, but less strong than in rat8-2 cells. Fluorescence microscopy images of in situ hybridization experiments with Cy3-labeled probes against the 25S rRNA are displayed for WT, rat7ΔN and rat8-2 cells upon shift for 1 h to 37°C. (TIF) [file pone.0149571.s005.tif]

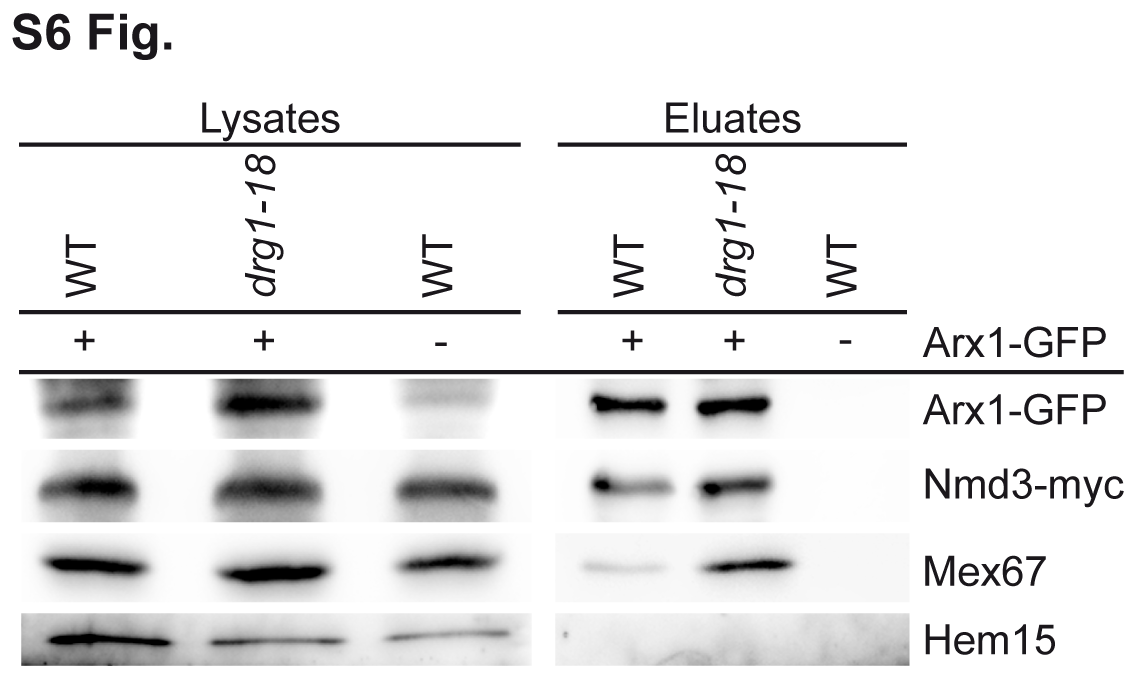

Supplement: S6 Fig — Western blot analyses of Arx1-GFP immunoprecipitations show an increased co-precipitation of Mex67 and Nmd3-myc in drg1-18 compared to wild type cells upon temperature shift for 1 h to 37°C. Detection of Hem15 served as negative control. (TIF) [file pone.0149571.s006.tif]

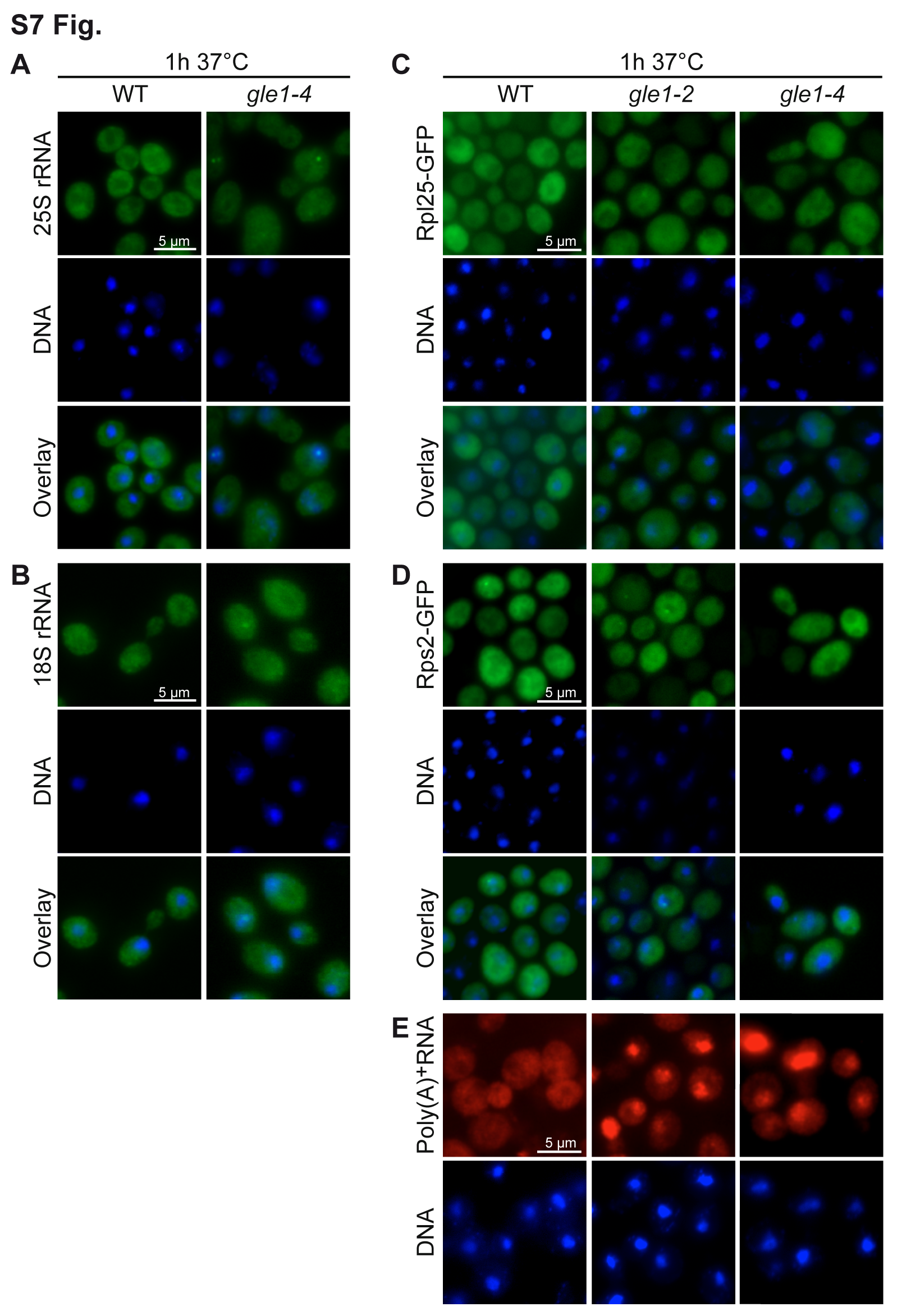

Supplement: S7 Fig — (A-B) Fluorescence in situ hybridization experiments with gle1-4 and wild type cells upon temperature shifts for 1 h to 37°C with probes against the 25S rRNA (A) and the 18S rRNA (B) are shown. (C-D) Fluorescence microscopy images of the ribosomal reporter proteins Rpl25-GFP (C) and Rps2-GFP (D) are shown in gle1-2, gle1-4 and wild type cells shifted for 1 h to 37°C. (E) Poly(A)+RNA accumulates in the nuclei of the different gle1 mutants. In situ hybridization experiments with Cy3-labeled oligo(dT)50 probes are shown in gle1-2, gle1-4 and wild type cells upon 1 h shift to 37°C. (TIF) [file pone.0149571.s007.tif]

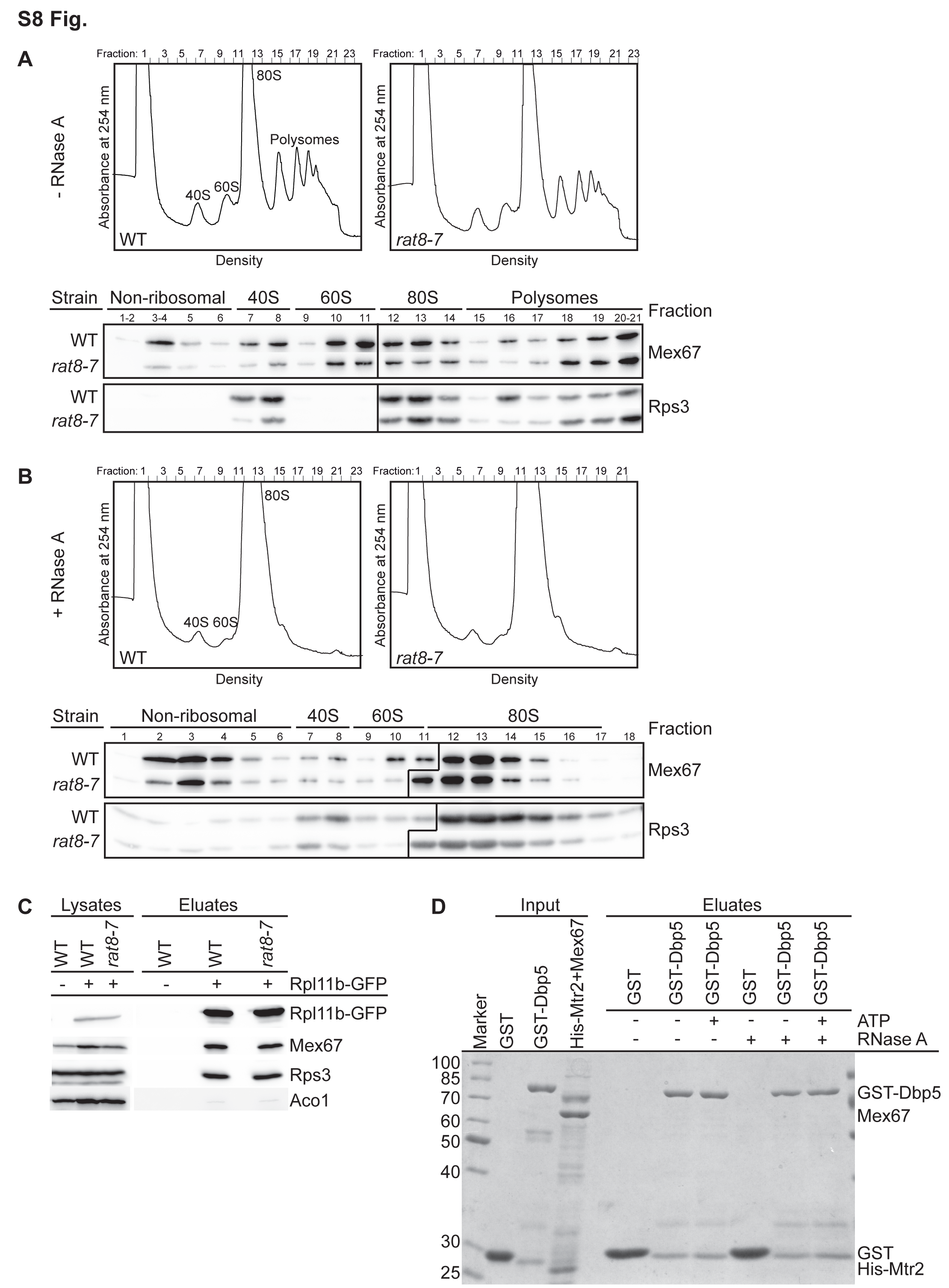

Supplement: S8 Fig — (A-B) Mex67 is associated with ribosomal fractions of sucrose-density gradients from wild type and rat8-7 cells without (A) and with (B) the addition of RNase A. Upper panels show flow through photometry (A254nm) profiles of wild type and rat8-7 cells shifted for 1 h to 16°C. Bottom panels show the corresponding separated protein fractions in Western blot analyses with direct antibodies against Mex67 and the ribosomal protein Rps3. (C) The interaction between the large ribosomal protein Rpl11b and Mex67 is not altered in the rat8-7 strain. Western blot analyses show co-precipitated Mex67 and as a positive control Rps3 in the Rpl11b-GFP immunoprecipitation after RNase A treatment. The protein level of Mex67 is not changed in rat8-7 cells compared to wild type upon 1 h temperature shifts to 16°C. Aco1 served as a non-binding control. (D) Dbp5 and Mex67 directly interact with each other. The Coomassie stained gel with the same samples of Fig 8B shows successful pull-down of GST-Dbp5 and GST with similar efficiency. The protein sizes are indicated in kDa. (TIF) [file pone.0149571.s008.tif]

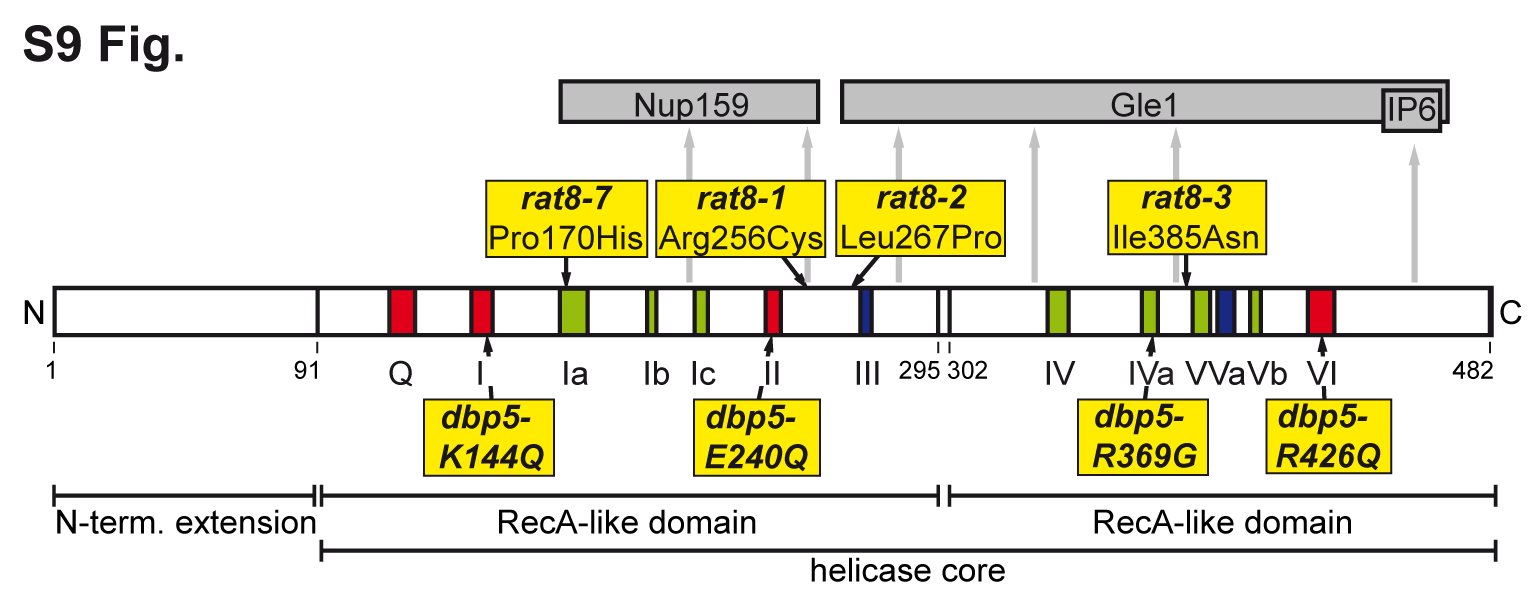

Supplement: S9 Fig — The scheme shows the 13 conserved sequence motifs that bind RNA (green), bind and hydrolyze ATP (red) or are necessary for both (blue). The positions of the amino acid substitutions in the different temperature-sensitive (at the top) and ATPase-deficient (at the bottom) dbp5/rat8 mutants are indicated in yellow. The co-factors Gle1, IP6 and Nup159 interact with the protein surface and important interaction sites are marked in gray. Picture modified from [16]. (TIF) [file pone.0149571.s009.tif]

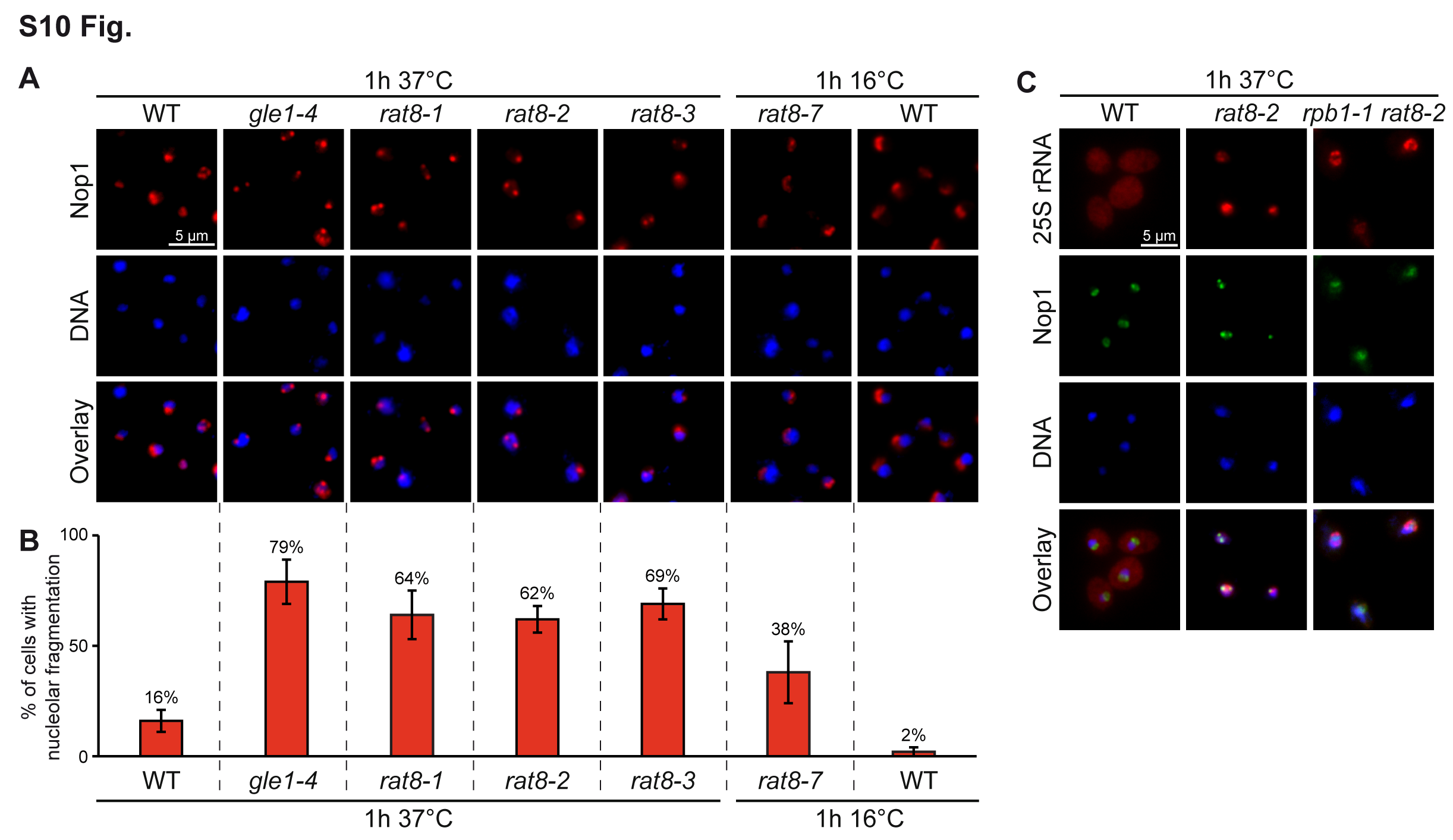

Supplement: S10 Fig — (A) Fluorescence microscopy images of immunofluorescence experiments to stain Nop1 as nucleolar marker show the intensity of nulceolar fragmentation in gle1-4, dbp5 mutants and wild type cells upon 1 h shift to their non-permissive temperatures. (B) Quantification of (A) displays the percentage of cells with nucleolar fragmentation. (C) The double mutant rpb1-1 rat8-2 shows less nucleolar fragmentation than rat8-2, but still nuclear accumulation of 25S rRNAs. Fluorescence microscopy images of combined in situ hybridization with Cy3-labeled 25S probes and immunofluorescence against the nucleolar protein Nop1 are shown in rpb1-1 rat8-2, rat8-2 and wild type cells upon shifts for 1 h to 37°C. (TIF) [file pone.0149571.s010.tif]

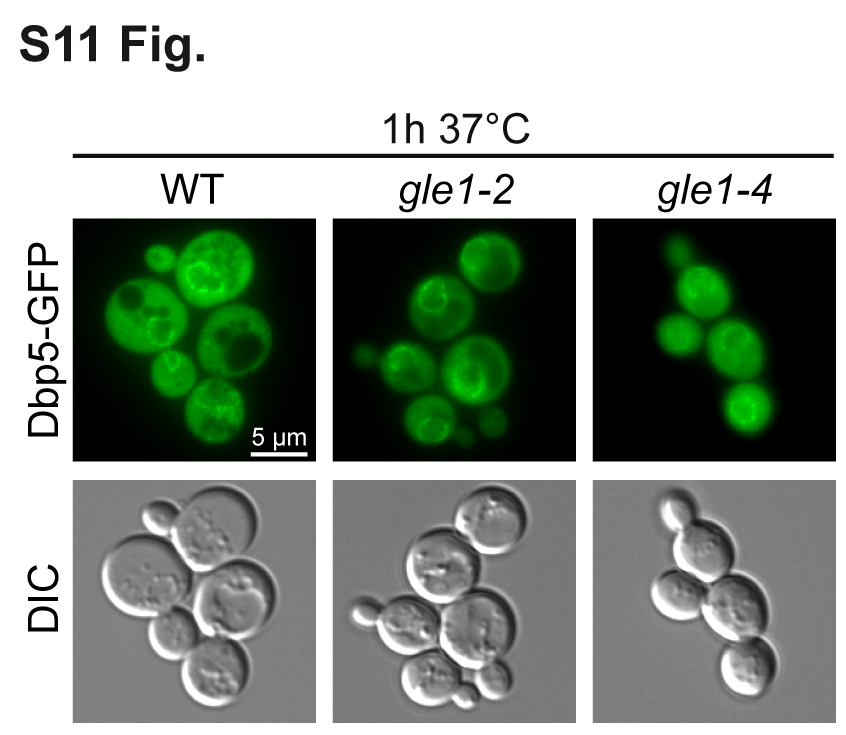

Supplement: S11 Fig — Fluorescence microscopy images of GFP-Dbp5 in living yeast cells are shown in gle1-2, gle1-4 and wild type cells upon 1 h shift to 37°C. (TIF) [file pone.0149571.s011.tif]

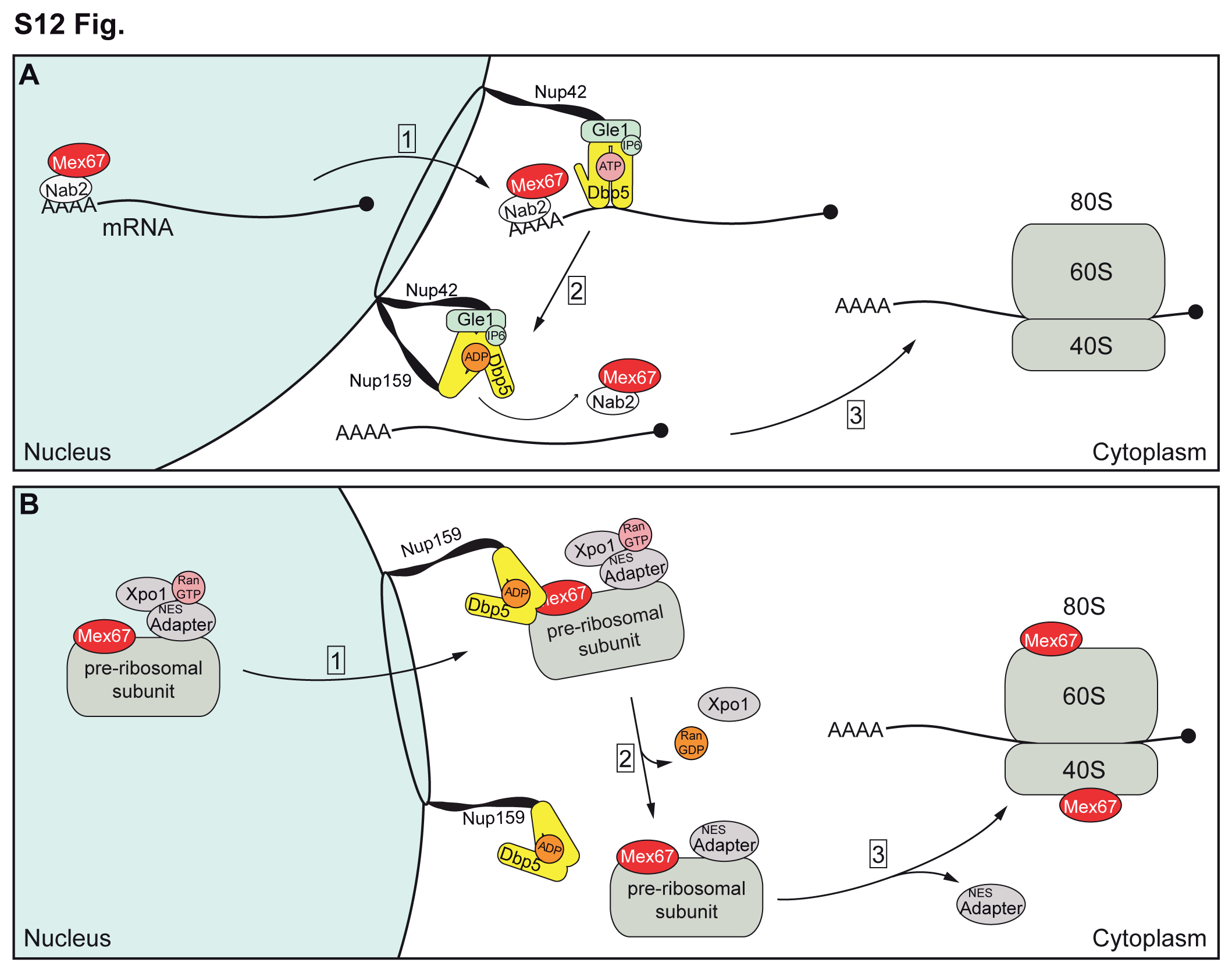

Supplement: S12 Fig — (A) Model for the role of Dbp5 in mRNA transport from the nucleus into the cytoplasm. The ATPase cycle of Dbp5 and its regulation by its cofactors is necessary to displace export factors such as Mex67 and Nab2 from the mRNA and to provide directionality. (B) Model for the function of Dbp5 in the nuclear export of both pre-ribosomal subunits. Dbp5 does not release Mex67 from the emerging ribosomal particles and the ATPase cycle of Dbp5 is dispensable for ribosomal transport. However, the presence of Dbp5 at the cytoplasmic side of the NPC is required, possibly to capture the export factor-bound pre-ribosomal particles and prevent their backsliding into the NPC. In this way, Dbp5 could support other export factors such as Xpo1 that generate directionality to the transport process by their RanGTP-hydrolysis induced dissociation. (TIF) [file pone.0149571.s012.tif]
