# Supplementary material for: Nuclear Export of Pre-Ribosomal Subunits Requires Dbp5, but Not as an RNA-Helicase as for mRNA Export
Source: PLoS One. 2016 Feb 12;11(2):e0149571. doi: 10.1371/journal.pone.0149571 (PMC4752221; doi:10.1371/journal.pone.0149571)
Supplement: S1 Table — (PDF) [file pone.0149571.s013.pdf]

S1 Table. *Saccharomyces cerevisiae* strains used in this study.

Strain number	Genotype	Source
HKY36	*MATα ura3-52 leu2Δ1 his3Δ200*	[1]
HKY40	*MAT ura3-1 leu2-3 trp1-1 his3-11 ade2-1 xpo1::LEU2 + pCEN XPO1 HIS3*	[2]
HKY41	*MATα ura3-1 leu2-3 trp1-1 his3-11 ade2-1 can1-1 xpo1::LEU2 + pCEN xpo1-1 HIS3*	[2]
HKY90	*MATa ura2 leu2 rat8-1*	this study, based on [3]
HKY93	*MATα ura3-1 leu2-3,112 his3-11 ade2-1 gle1-4*	[4]
HKY124	*MATα ura3-52 leu2Δ1 his3Δ200 rat7-1*	[5]
HKY128	*MATα ura3-52 leu2Δ1 trp1Δ63 his3Δ200 rat8::HIS3 + pCEN rat8-3 LEU2*	[3]
HKY129	*MATa ura3-52 leu2Δ1 trp1Δ63 his3Δ200 rat8::HIS3 + pCEN rat8-7 LEU2*	[3]
HKY130	*MATa ura3-52 leu2Δ1 trp1Δ63 rat8-2*	[3]
HKY206	*MATα ura3-1 leu2-3 trp1-1 his3-11 ade2-1 can1-1 xpo1::Leu2 + pCEN xpo1-1 TRP1*	[6]
HKY276	*MATa ade2 his3 leu2 trp1 ura3*	[7]
HKY314	*MATa ura3Δ0 leu2Δ0 his3Δ1 met15Δ0*	Euroscarf
HKY381	*MATα ura3Δ0 leu2Δ0 his3Δ1 lys2Δ0*	Euroscarf
HKY456	*MATa ura3-52 leu2Δ1 trp1Δ63 his3Δ200 rat8::HIS3 + p 2µ RAT8 URA3*	this study
HKY462	*MATα ura3-52 leu2Δ1 rpb1-1 rat8-2*	this study
HKY734	*MATα ura3Δ0 leu2Δ0 his3Δ1 lys2Δ mtr2::kanMX4 + pCEN mtr2-33 LEU2*	this study
HKY863	*MATα ura3Δ0 leu2Δ0 his3Δ1 lys2Δ rpl10::kanMX4 + pCEN rpl10(G161D)-GFP URA3*	[8]
HKY892	*MATa ura3 leu2 trp1 his3 ade2 mtr2::HIS3 + pCEN mtr2-33 TRP1 + pCEN MTR2 URA3*	[9]
HKY894	*MATα ura3 leu2 trp1 his3 lys2 nmd3::kanMX4 + pCEN nmd3-2 TRP1 + pCEN NMD3 URA3*	[9]
HKY1071	*MATa ura3 leu2 trp1 ade2 lys2 gle1-2*	[10]
HKY1242	*MATa ura3Δ0 leu2Δ0 his3Δ1 met15Δ0 RAT8:GFP:HIS3MX6X*	Invitrogen
HKY1356	*MATa ura3 leu2 trp1 his3 nmd3::kanMX4 rat8::HIS3 + p 2µ RAT8 URA3 + pCEN nmd3-2 TRP1 + pCEN NMD3 URA3*	this study
HKY1369	*MATα ura3 leu2 trp1 his3 lys2 mtr2::kanMX4 rat8::HIS3 + pCEN rat8-3 LEU2 + pCEN MTR2 URA3*	this study
HKY1370	*MATa ura3 leu2 his3*	this study
HKY1371	*MATa ura3 leu2 trp1 his3 lys2 rat8::HIS3 + pCEN rat8-3 LEU2*	this study
HKY1372	*MATα ura3 leu2 his3 mtr2::kanMX4 + pCEN mtr2-33 LEU2 + pCEN MTR2 URA3*	this study
HKY1377	*MATa ura3Δ0 leu2Δ0 his3Δ1 met15Δ0 XPO1:GFP:HIS3MX6X*	Invitrogen
HKY1379	*MATa ura3Δ0 leu2Δ0 his3Δ1 met15Δ0 RIO2:GFP:HIS3MX6X*	Invitrogen
HKY1493	*MATa ade2 ura3-0 his3-1 leu2-0 trp1 drg1-18*	[11]
HKY1646	*MATa ura3-52 leu2Δ1 trp1Δ63 his3Δ200 rat7ΔN*(1-500)	[12]

Supplemental References

1. Winston F, Dollard C, Ricupero-Hovasse SL. Construction of a set of convenient Saccharomyces cerevisiae strains that are isogenic to S288C. Yeast. 1995;11(1):53-5. Epub 1995/01/01. doi: 10.1002/yea.320110107. PubMed PMID: 7762301.

2. Stade K, Ford CS, Guthrie C, Weis K. Exportin 1 (Crm1p) is an essential nuclear export factor. Cell. 1997;90(6):1041-50. Epub 1997/10/10. doi: S0092-8674(00)80370-0 [pii]. PubMed PMID: 9323132.
3. Snay-Hodge CA, Colot HV, Goldstein AL, Cole CN. Dbp5p/Rat8p is a yeast nuclear pore-associated DEAD-box protein essential for RNA export. EMBO J. 1998;17(9):2663-76. Epub 1998/06/20. doi: 10.1093/emboj/17.9.2663. PubMed PMID: 9564048; PubMed Central PMCID: PMC1170607.
4. Murphy R, Wente SR. An RNA-export mediator with an essential nuclear export signal. Nature. 1996;383(6598):357-60. Epub 1996/09/26. doi: 10.1038/383357a0. PubMed PMID: 8848052.
5. Del Priore V, Heath C, Snay C, MacMillan A, Gorsch L, Dagher S, et al. A structure/function analysis of Rat7p/Nup159p, an essential nucleoporin of Saccharomyces cerevisiae. J Cell Sci. 1997;110 (Pt 23):2987-99. Epub 1998/02/07. PubMed PMID: 9359887.
6. Taura T, Krebber H, Silver PA. A member of the Ran-binding protein family, Yrb2p, is involved in nuclear protein export. Proc Natl Acad Sci U S A. 1998;95(13):7427-32. Epub 1998/06/24. PubMed PMID: 9636166; PubMed Central PMCID: PMC22639.
7. Zenklusen D, Vinciguerra P, Strahm Y, Stutz F. The yeast hnRNP-Like proteins Yra1p and Yra2p participate in mRNA export through interaction with Mex67p. Mol Cell Biol. 2001;21(13):4219-32. Epub 2001/06/08. doi: 10.1128/MCB.21.13.4219-4232.2001. PubMed PMID: 11390651; PubMed Central PMCID: PMC87083.
8. Baierlein C, Hackmann A, Gross T, Henker L, Hinz F, Krebber H. Monosome formation during translation initiation requires the serine/arginine-rich protein Npl3. Mol Cell Biol. 2013;33(24):4811-23. Epub 2013/10/09. doi: 10.1128/MCB.00873-13
MCB.00873-13 [pii]. PubMed PMID: 24100011; PubMed Central PMCID: PMC3889561.
9. Bassler J, Grandi P, Gadal O, Lessmann T, Petfalski E, Tollervey D, et al. Identification of a 60S preribosomal particle that is closely linked to nuclear export. Mol Cell. 2001;8(3):517-29. Epub 2001/10/05. doi: S1097-2765(01)00342-2 [pii]. PubMed PMID: 11583615.
10. Bolger TA, Folkmann AW, Tran EJ, Wente SR. The mRNA export factor Gle1 and inositol hexakisphosphate regulate distinct stages of translation. Cell. 2008;134(4):624-33. Epub 2008/08/30. doi: S0092-8674(08)00779-4 [pii]
10.1016/j.cell.2008.06.027. PubMed PMID: 18724935; PubMed Central PMCID: PMC2601711.
11. Zakalskiy A, Hogenauer G, Ishikawa T, Wehrschutz-Sigl E, Wendler F, Teis D, et al. Structural and enzymatic properties of the AAA protein Drg1p from Saccharomyces cerevisiae. Decoupling of intracellular function from ATPase activity and hexamerization. J Biol Chem. 2002;277(30):26788-95. Epub 2002/05/15. doi: 10.1074/jbc.M201515200. PubMed PMID: 12006565.
12. Scarcelli JJ, Hodge CA, Cole CN. The yeast integral membrane protein Apq12 potentially links membrane dynamics to assembly of nuclear pore complexes. J Cell Biol. 2007;178(5):799-812. Epub 2007/08/29. doi: 10.1083/jcb.200702120. PubMed PMID: 17724120; PubMed Central PMCID: PMC2064545.
