# Supplementary material for: Nuclear Export of Pre-Ribosomal Subunits Requires Dbp5, but Not as an RNA-Helicase as for mRNA Export
Source: PLoS One. 2016 Feb 12;11(2):e0149571. doi: 10.1371/journal.pone.0149571 (PMC4752221; doi:10.1371/journal.pone.0149571)
Supplement: S2 Table — (PDF) [file pone.0149571.s014.pdf]

S2 Table. Plasmids used in this study.

Plasmid number	Features	Source
pHK20	*CEN MEX67-GFP LEU2*	[1]
pHK43	*CEN XPO1-GFP URA3*	[2]
pHK86	*CEN TRP1*	[3]
pHK87	*CEN LEU2*	[3]
pHK88	*CEN URA3*	[3]
pHK258	*2μ P_{GAL1}CBP80-GFP URA3*	[4]
pHK284	*CEN MTR2 URA3*	[1]
pHK636	*2μ P_{GAL}MYC-DBP5 LEU2*	this study
pHK638	*CEN rat8-3 LEU2*	[5]
pHK653	*CEN GFP-DBP5 URA3*	[6]
pHK655	*CEN GFP-rat8-2 LEU2*	[6]
pHK666	*CEN GFP-DBP5 LEU2*	this study
pHK697	*CEN RPS2-GFP URA3*	[7]
pHK698	*CEN RPL25-GFP URA3*	[8]
pHK706	*CEN mtr2-33 TRP1*	[9]
pHK707	*CEN mtr2-33 LEU2*	[9]
pHK720	*CEN NMD3-13xmyc LEU2*	[10]
pHK724	*CEN NMD3-GFP URA3*	[11]
pHK789	*CEN ARX1-GFP URA3*	this study
pHK1288	pGEX-6P-1 (expression plasmid for GST)	GE Healthcare
pHK1289	pGEX-6P-1-*DBP5* (expression plasmid for GST-Dbp5)	this study
pHK1349	*CEN RPL11B-GFP URA3*	this study
pHK1372	pProEX-1-*MTR2*-RBS-*MEX67*<br>(expression plasmid for His-Mtr2 and Mex67)	[12]
pHK1398	*2μ P_{GAL}dbp5-R369G LEU2*	[13]
pHK1399	*2μ P_{GAL}dbp5-R426Q LEU2*	[13]
pHK1508	*2μ P_{GAL}dbp5-E240Q LEU2*	[13]
pHK1509	*2μ P_{GAL}dbp5-K144Q LEU2*	[13]

Supplemental References

1. Santos-Rosa H, Moreno H, Simos G, Segref A, Fahrenkrog B, Pante N, et al. Nuclear mRNA export requires complex formation between Mex67p and Mtr2p at the nuclear pores. Mol Cell Biol. 1998;18(11):6826-38. Epub 1998/10/17. PubMed PMID: 9774696; PubMed Central PMCID: PMC109266.
2. Stade K, Ford CS, Guthrie C, Weis K. Exportin 1 (Crm1p) is an essential nuclear export factor. Cell. 1997;90(6):1041-50. Epub 1997/10/10. doi: S0092-8674(00)80370-0 [pii]. PubMed PMID: 9323132.
3. Sikorski RS, Hieter P. A system of shuttle vectors and yeast host strains designed for efficient manipulation of DNA in Saccharomyces cerevisiae. Genetics. 1989;122(1):19-27. Epub 1989/05/01. PubMed PMID: 2659436; PubMed Central PMCID: PMC1203683.
4. Shen EC, Stage-Zimmermann T, Chui P, Silver PA. 7The yeast mRNA-binding protein Npl3p interacts with the cap-binding complex. J Biol Chem. 2000;275(31):23718-24. Epub 2000/05/24. doi: 10.1074/jbc.M002312200. PubMed PMID: 10823828.

5. Snay-Hodge CA, Colot HV, Goldstein AL, Cole CN. Dbp5p/Rat8p is a yeast nuclear pore-associated DEAD-box protein essential for RNA export. EMBO J. 1998;17(9):2663-76. Epub 1998/06/20. doi: 10.1093/emboj/17.9.2663. PubMed PMID: 9564048; PubMed Central PMCID: PMC1170607.
6. Gross T, Siepmann A, Sturm D, Windgassen M, Scarcelli JJ, Seedorf M, et al. The DEAD-box RNA helicase Dbp5 functions in translation termination. Science. 2007;315(5812):646-9. Epub 2007/02/03. doi: 315/5812/646 [pii] 10.1126/science.1134641. PubMed PMID: 17272721.
7. Milkereit P, Strauss D, Bassler J, Gadal O, Kuhn H, Schutz S, et al. A Noc complex specifically involved in the formation and nuclear export of ribosomal 40 S subunits. J Biol Chem. 2003;278(6):4072-81. Epub 2002/11/26. doi: 10.1074/jbc.M208898200. PubMed PMID: 12446671.
8. Gadal O, Strauss D, Kessl J, Trumpower B, Tollervey D, Hurt E. Nuclear export of 60s ribosomal subunits depends on Xpo1p and requires a nuclear export sequence-containing factor, Nmd3p, that associates with the large subunit protein Rpl10p. Mol Cell Biol. 2001;21(10):3405-15. Epub 2001/04/21. doi: 10.1128/MCB.21.10.3405-3415.2001. PubMed PMID: 11313466; PubMed Central PMCID: PMC100262.
9. Bassler J, Grandi P, Gadal O, Lessmann T, Petfalski E, Tollervey D, et al. Identification of a 60S preribosomal particle that is closely linked to nuclear export. Mol Cell. 2001;8(3):517-29. Epub 2001/10/05. doi: S1097-2765(01)00342-2 [pii]. PubMed PMID: 11583615.
10. Ho JH, Kallstrom G, Johnson AW. Nmd3p is a Crm1p-dependent adapter protein for nuclear export of the large ribosomal subunit. J Cell Biol. 2000;151(5):1057-66. Epub 2000/11/22. PubMed PMID: 11086007; PubMed Central PMCID: PMC2174350.
11. Hedges J, West M, Johnson AW. Release of the export adapter, Nmd3p, from the 60S ribosomal subunit requires Rpl10p and the cytoplasmic GTPase Lsg1p. EMBO J. 2005;24(3):567-79. Epub 2005/01/22. doi: 7600547 [pii] 10.1038/sj.emboj.7600547. PubMed PMID: 15660131; PubMed Central PMCID: PMC548654.
12. Yao W, Roser D, Kohler A, Bradatsch B, Bassler J, Hurt E. Nuclear export of ribosomal 60S subunits by the general mRNA export receptor Mex67-Mtr2. Mol Cell. 2007;26(1):51-62. Epub 2007/04/17. doi: S1097-2765(07)00118-9 [pii] 10.1016/j.molcel.2007.02.018. PubMed PMID: 17434126.
13. Hodge CA, Tran EJ, Noble KN, Alcazar-Roman AR, Ben-Yishay R, Scarcelli JJ, et al. The Dbp5 cycle at the nuclear pore complex during mRNA export I: dbp5 mutants with defects in RNA binding and ATP hydrolysis define key steps for Nup159 and Gle1. Genes Dev. 2011;25(10):1052-64. Epub 2011/05/18. doi: 25/10/1052 [pii]
10.1101/gad.2041611. PubMed PMID: 21576265; PubMed Central PMCID: PMC3093121.
