# Supplementary material for: Nuclear Export of Pre-Ribosomal Subunits Requires Dbp5, but Not as an RNA-Helicase as for mRNA Export
Source: PLoS One. 2016 Feb 12;11(2):e0149571. doi: 10.1371/journal.pone.0149571 (PMC4752221; doi:10.1371/journal.pone.0149571)
Supplement: S3 Table — (PDF) [file pone.0149571.s015.pdf]

S3 Table. Oligonucleotides used in this study.

Oligo number	Sequence	Target
HK558	5’-tcc**CCGCGG**TGGGCTTCTCTAGCTC-3’	*ARX1* forward **(*Sac*II site)**
HK562	5’-GTTCCTCCCAGTGAG-3’	*ARX1* reverse
HK974	5'-**taatacgactcactatagggg**GCTTAAGTTCAGCGGGTACTCCTACC-3'	*RDN25* reverse **(T7 promoter)**
HK1138	5'-AGGTAGGAGTACCCGCTGAA-3'	*RDN25* forward
HK1139	5'-**taatacgactcactatagggg**ATGGAATTTACCACCCACTTAGAGC-3'	*RDN25* reverse **(T7 promoter)**
HK1140	5'-GTGAAACTGCGAATGGCTCATTAAAT-3'	*RDN18* forward
HK1141	5'-**taatacgactcactatagggg**AATCGAACCCTTATTCCCCGTTA-3'	*RDN18* reverse **(T7 promoter)**
HK1485	5'-tat**CCGCGG**TGCCCAAGTCAAGCCTAC-3'	*RPL11B* forward **(*Sac*II site)**
HK1486	5'-cta**CTCGAG**cTTTATCGAGCACATCAGCG-3'	*RPL11B* reverse **(*Xho*I site)**
HK1723	5'-CGAGCCGTTTATGTCCAACG-3'	*LSR1* forward
HK1724	5'-**taataggactcactatagg**GCCGATACTTGGGGGATAAG-3'	*LSR1* reverse **(T7 promoter)**
HK1893	5'-GTAGGTGAACCTGCGGAAGG-3'	*RDN18* forward
HK1894	5'-**taatacgactcactatagggg**GCACAGAAATCTCTCACCGTTTG-3'	*ITS1* reverse **(T7 promoter)**
HK1895	5'-GCCTGTTTGAGCGTCATTTCCTTCTC-3'	*RDN5.8/ITS2* forward
HK2200	5'-GAACAGGACGTCATAGAGGGTGAGAATCCC GTGTGGCGAGGAGTGCGGTT[Cyanine3]-3’	*RDN25* probe
